# Perfluorophenyl Derivatives as Unsymmetrical Linkers for Solid Phase Conjugation

**DOI:** 10.3389/fchem.2018.00589

**Published:** 2018-11-28

**Authors:** Saba Alapour, Anamika Sharma, Beatriz G. de la Torre, Deresh Ramjugernath, Neil A. Koorbanally, Fernando Albericio

**Affiliations:** ^1^School of Chemistry and Physics, University of KwaZulu-Natal, Durban, South Africa; ^2^KRISP, College of Health Sciences, University of KwaZulu-Natal, Durban, South Africa; ^3^School of Chemical Engineering, University of KwaZulu-Natal, Durban, South Africa; ^4^Department of Organic Chemistry, CIBER-BBN (Networking Centre on Bioengineering, Biomaterials and Nanomedicine), University of Barcelona, Barcelona, Spain

**Keywords:** fluorolinkers, conjugation, solid-phase peptide synthesis, peptide stapling, chimera

## Abstract

Linkers play major roles in conjugation chemistry toward the advancement of drug discovery. Two different series of fluorinated linkers were introduced to the backbone of a model peptide using solid phase peptide synthesis. These fluorinated linkers have the potential to conjugate two asymmetrical groups. This has not been done using other fluorinated linkers. This study deals with application of linkers with S, N, and O terminals and reports on the investigation of their chemoselectivity and activity for branching peptide backbones using a chosen model peptide. These fluorinated linkers have unique properties that will make it possible for a large diversity of bioconjugated chemicals for different bioapplications to be designed and synthesized.

## Introduction

Conjugation of molecules is a chemical tool with increasing interest in several scientific areas, such as pharmaceuticals or materials. Several techniques have been reported in the literature, but many of them have drawbacks associated with chemoselectivity, tunability, and/or synthetic practicality (Schilling et al., 2011). On the other hand, the presence of fluorine in a molecule is key for fine-tuning its properties. The high electronegativity of fluorine has a number of obvious advantages leading to polarization and imparting a less covalent and more electrostatic character to the C–F bond. This leads to a relatively large dipole, which interacts with other dipoles in its vicinity, resulting in a conformational change (O'Hagan, 2008). Furthermore, the presence of fluorine in the backbone of an organic compound results in some changes such as omniphobicity/lipophilicity and electrostatic interactions that can dramatically influence its chemical reactivity (O'Hagan, 2008; Wang et al., 2014). Furthermore, one of the important effects of fluorination is the alteration of acidity and basicity of the parent compounds (Kobzev et al., 1989; van Niel et al., 1999; Rowley et al., 2001; Morgenthaler et al., 2007). In the pharmaceutical field, this can intensely influence binding affinity, pharmacokinetic properties, and bioavailability of a given drug candidate. More importantly, it must be emphasized that fluorine provides significant impact in pharmaceuticals in general, not only on fluorinated drugs but also in various health care products. Fluorine scanning is currently a routine approach for the development of novel fluorinated drug candidates. It is quite remarkable that three out of the five top-selling pharmaceuticals contain a fluorine atom in its structure (Wang et al., 2014).

**Scheme 1:**
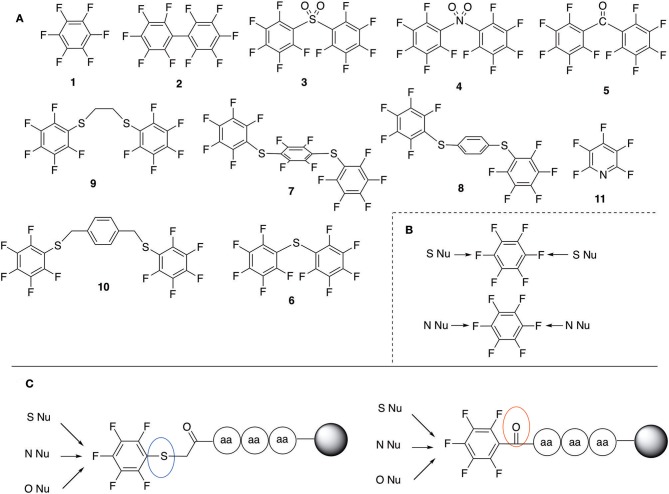
**(A)** Perfluoro aromatic linkers that has been previously investigated **(B)** Types of S_N_Ar nucleophilic reactions **(C)** Present work.

Pentelute and co-workers have extensively studied the use of perfluororoaryl linkers for different applications in the peptide field, such as macrocyclization and/or peptide stapling as well as site-selective bioconjugation (Spokoyny et al., 2013; Zhang et al., 2013, 2014, 2016; Zou et al., 2014; Dai et al., 2016; Lautrette et al., 2016; Lühmann et al., 2016; Evans and Pentelute, 2018). Scheme 1A shows some examples of fluorine containing linkers such as perfluorobenzene (1), perfluorobiphenyl (2), and other fluorinated linkers that have been applied and studied in bioconjugation chemistry (Hynes and Peach, 1986; Spokoyny et al., 2013; Zhang et al., 2013, 2014; Zou et al., 2014; Dai et al., 2016; Lautrette et al., 2016; Kalhor-Monfared et al., 2017; Evans and Pentelute, 2018).

All of the linkers previously described are symmetric (Scheme 1B), which might limit their application to some extent. To the best of our knowledge, all of the reported fluorine-based linkers are connecting mainly through S and in much less extension N nucleophiles in their/two sides. Finally, all fluorine based conjugation has been carried out in solution, which could be less selective and require tedious purification of the final products (Spokoyny et al., 2013; Zhang et al., 2013, 2014; Zou et al., 2014; Dai et al., 2016; Lautrette et al., 2016; Kalhor-Monfared et al., 2017; Evans and Pentelute, 2018). Herein, we report on the investigation of unsymmetrical fluorine based linkers in solid phase that enable peptides to be coupled to N, O, and S containing substituents (Scheme 1C).

This study investigates the reactivity and site specificity of two new generation of perfluoroaromatic linkers, 2–6-pentafluoroacetic acid and 2–6-pentafluorothiophenol anchored to the model peptide H-Gly-Phe-Leu-NH-resin (Scheme 1C). Both linkers are commercially available, which could facilitate its use by the scientific community. The reactivity of these novel scaffolds/linkers under S_N_Ar with three types of soft-hard nucleophiles such as amines, thiols and alcohols was studied, which represent the functionality found in peptides and other biomolecules. Overall, an unsymmetrical substitution in these fluorine containing linkers is illustrated.

## Results and discussion

**Scheme 2:**
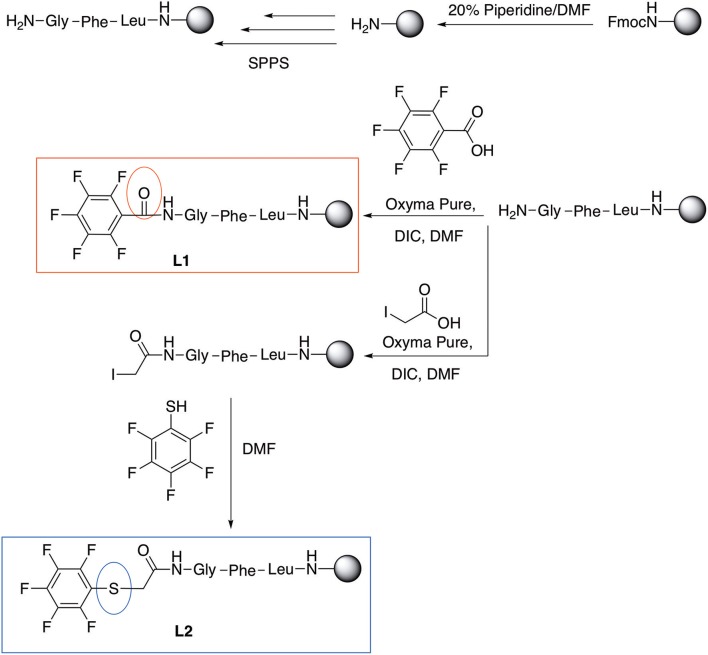
Scheme for the preparation of the short peptide and two different linkers **L**_1_ and **L**_2_.

In addition to the pentafluoro system, the first bidentate linker contains a carboxylic acid, while the second a thiol (Scheme 2), which results in diversity in the conjugation. In the first instance, the short peptide H-Gly-Phe-Leu-NH-Resin was built up using solid-phase peptide synthesis (SPPS) methodology. 2–6-Pentafluorobenzoic acid was safely coupled to the tripeptidyl resin using DIC and OxymaPure to form **L**_1_. On the other hand, 2–6-pentafluorothiophenol was incorporated to the iodoacetyl derivative of the tripeptidyl resin in the absence of any extra reagent to form **L**_2_ (Scheme 2). **L**_1_ contained a carboxamide which is an electron withdrawing group and **L**_2_ an alkylthio S which is an electron donating.

**Figure 1:**
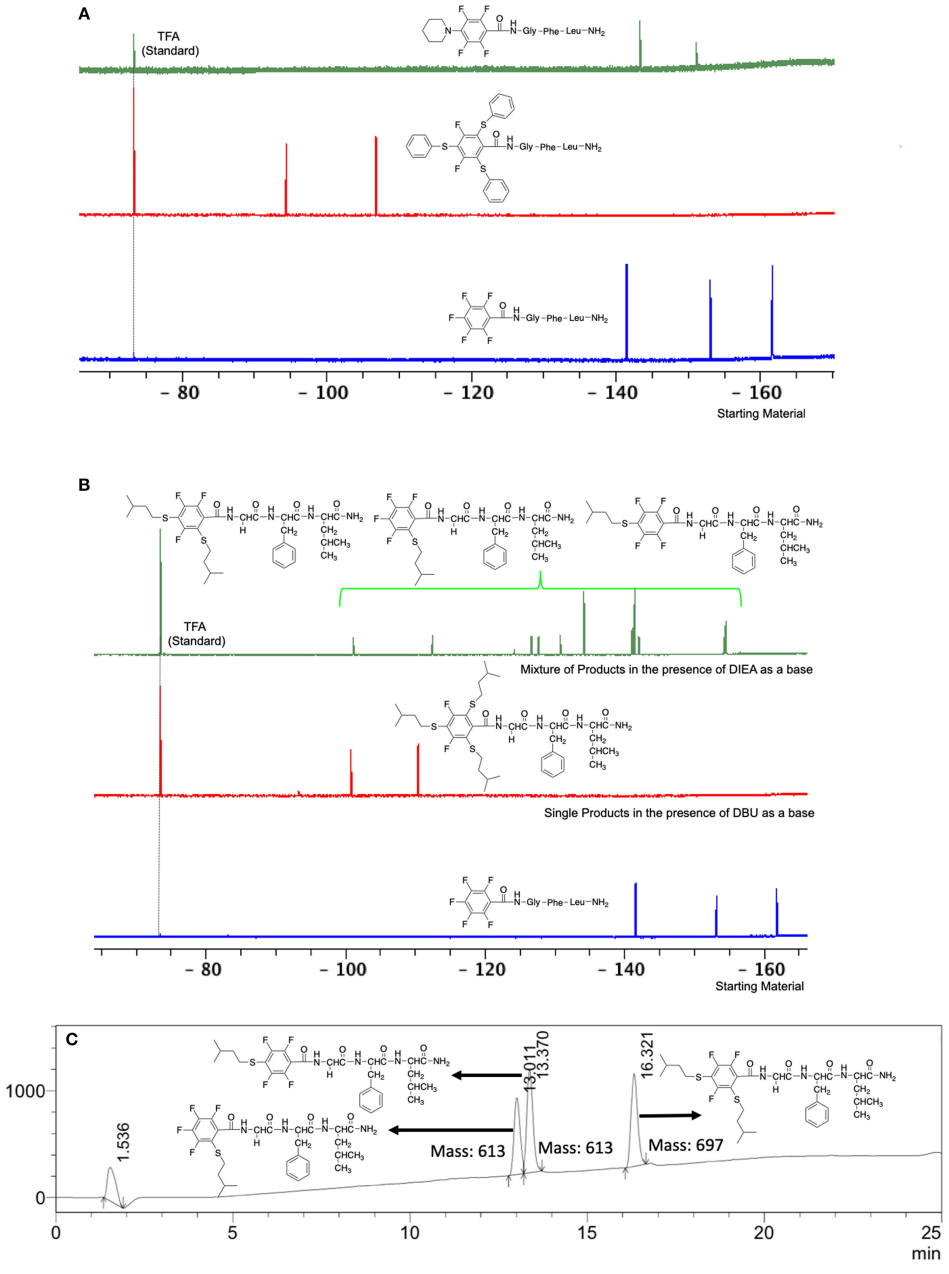
**(A)** Comparison of the ^19^F NMR of the product of the reaction between piperidine and benzenethiol as a reactant with the peptide linker **L**_1_ using DBU as a base and NMP as solvent (room temp., 1 h) and the starting material **L**_1_; **(B)**. Comparison of ^19^F NMR of the product of the reaction of 3-methyl-1-butanethiol with starting material **L**_1_ in two different bases (DBU and DIEA) using NMP as a solvent (room temp., 1 h); **(C)**. LC-MS of the reaction of methyl-1-butanethiol with the starting material **L**_1_ in NMP and DIEA as a base (acetonitrile:water step gradient from 30 to 95% in 15 min and held at 95% acetonitrile in water for 5 min, then flushed with 30% acetonitrile in water for 5 min).

The reactivity of these two series of peptidyl resins in S_N_Ar reactions with different nucleophiles were investigated. Both peptidyl resins were left to react with thiols, amines, phenols, and alcohols, which are the nucleophiles present in the biomolecules. Firstly, using butylamine, which has a moderate reactivity in comparison to S nucleophiles, solvent optimization was studied using *N,N'-*diisopropylethylamine (DIEA) as a base and **L**_1_ as substrate. The use of polar solvents such *N,N-*dimethylformamide (DMF) and *N-*methylpyrrolidone (NMP) results in a higher yield when compared to less polar solvents such as dichloromethane (DCM) and/or toluene (Table 1). This is possibly due to stabilization of the Meinsheimer complex formed during the S_N_Ar (Alapour et al., 2015). The best yield obtained during solvent optimization was 35% in NMP (#2, Table 1). This can be considered mediocre. Poor conversion was also experienced when Tris was used as a base. However, use of the non-nucleophilic DBU, a stronger base than DIEA, resulted in yields of 99%. Thus, the optimized conditions were identified as using NMP as a solvent and DBU as a base with a relatively short reaction time of 1 h (#6, Table 1). In all cases, ^19^F NMR confirmed that the regioisomer *para* to the carboxamide group was the only product obtained (Table 2, Figure 1A).

**Table 1:** Investigation of Optimized condition.

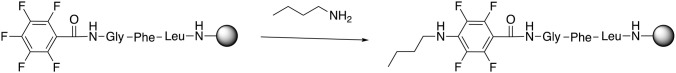				
**#**	**Solvent**	**Base**	**Reaction condition**	**Yield**^a^ **(%)**
1	DMF	DIEA	RT	30
2	NMP	DIEA	RT	35
3	DCM	DIEA	RT	5
4	Toluene	DIEA	RT	–
5	NMP	Tris	RT	20
6	NMP	DBU	RT	99

a *The yields are calculated using HPLC*.

**Table 2:** Application of the optimized conditions in nucleophilic reactions.

**#**	**Peptide**	**Nucleophile**	**Substitution**	**Yield**	**Reaction time (h)**
1	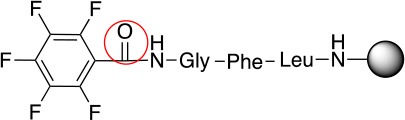	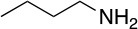	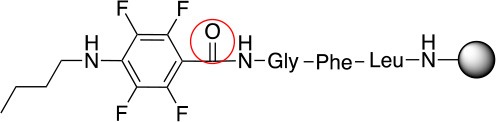	99	1
2	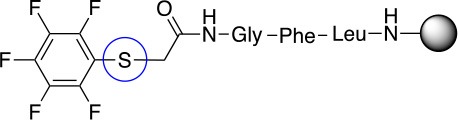	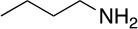	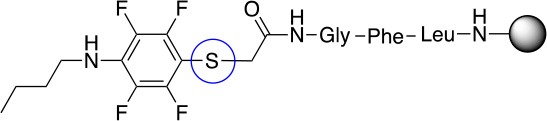	99	1
3	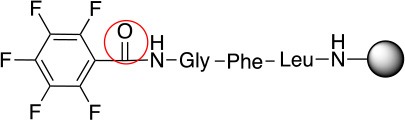	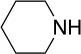	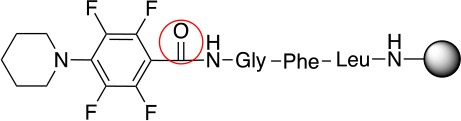	99	1
4	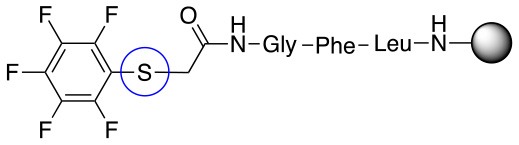	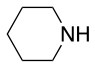	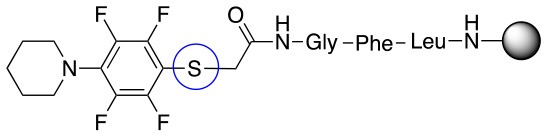	99	1
5	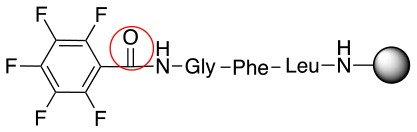	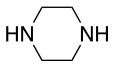	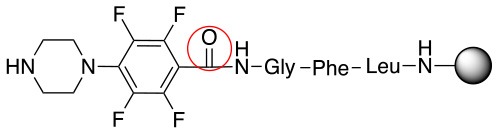	99	1
6	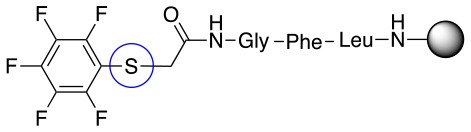	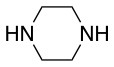	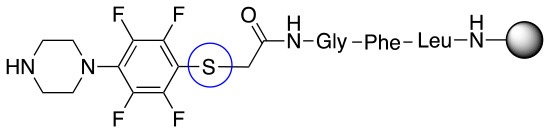	99	1
7	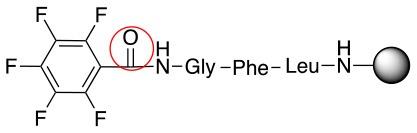	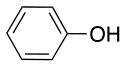	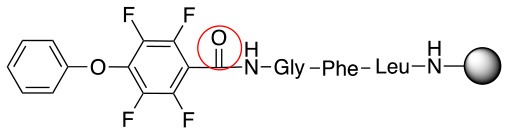	99	24
8	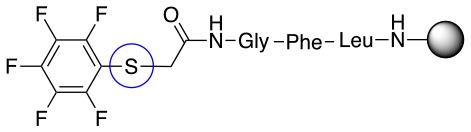	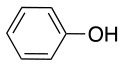	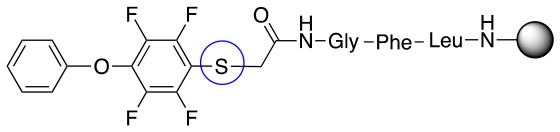	99	24
9	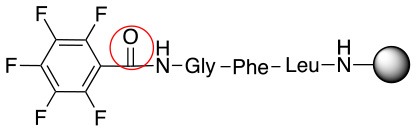	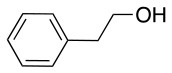	–	No reaction	48
10	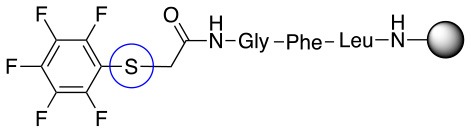	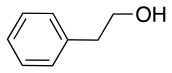	–	No reaction	48
11	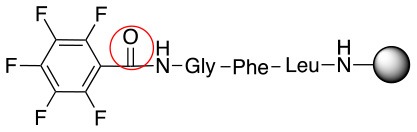	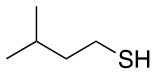	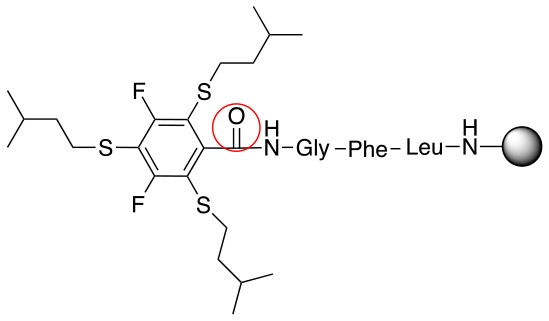	99	1
12	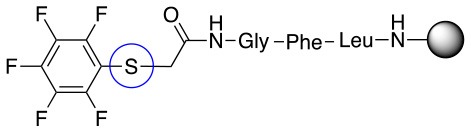	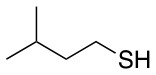	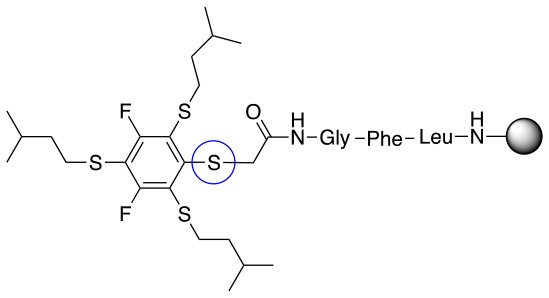	99	1
13	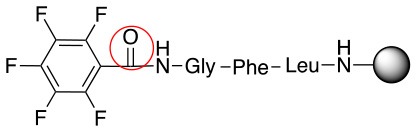	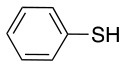	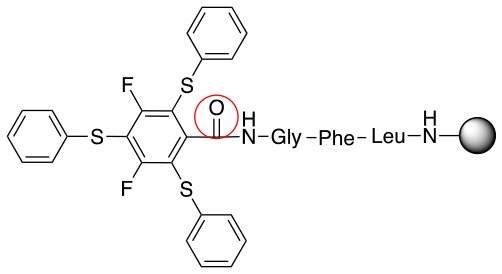	99	1
14	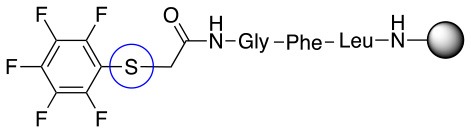	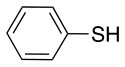	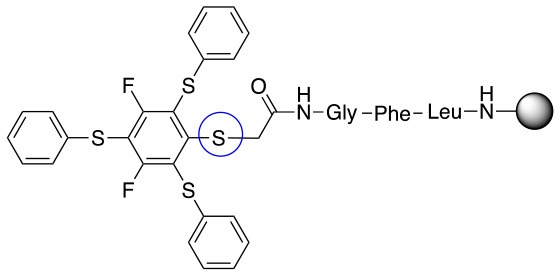	99	1

*Reagents and base are added 10 times in excess*.

The same conditions (NMP as a solvent and DBU as a base) were used for **L**_2_ with the same high yields (#2, Table 2). Piperidine and piperazine as nucleophiles also showed excellent conversion in all cases in just 1 h (Table 2, #1–6). Selectivity of the reaction toward *para* substitution was not affected when a secondary amine was used (Table 2, #3–6). Furthermore, when the bis-amine (piperazine) was used, cross-linking was not detected (Table 2, #5–6).

The reaction with phenol was slower as full conversion was only reached after 24 h. In both cases, only the substitution in *para* position was detected (Table 2, #7 and 8). On the other hand, no product was observed with aliphatic alcohols (ROH), even after 48 h (Table 2, #9 and 10).

Finally, both aliphatic and aromatic S containing nucleophiles resulted in triple substitutions in both *ortho* and *para* positions (Table 2, #11–14). Stabilization of the Meisenheimer complex through resonance of the negative charge at the sulfur on the *para* position of the thioether moiety enhanced reactivity of the linker-peptide complex to nucleophilic substitution (Birchall et al., 1967; Langille and Peach, 1972; Zou et al., 2014; Alapour et al., 2018). In this case, this reactivity was observed by substitution up to three positions in both groups of I and II.

A milder base, DIEA was also applied in the investigation of the reaction of 3-methylbutan-1-thiol and **L**_1_ for S substitution. However, a mixture of mono-*ortho*, mono-*para* and di-*para* and *ortho* products were obtained. This was confirmed via ^19^F NMR and LCMS and can be seen in Figure 1B and Figure 1C, respectively. Using a stronger base, DBU, only a single trisubstituted product at the *ortho* and *para* positions were observed (Figure 1B). However, the use of DIEA as a weak base for this fast S_N_Ar allowed us to have a better understanding of the present sequence in this reaction. According to the HPLC profile, it seems that strong nucleophiles such as RSH starts with monosubstitution at either the *para* or *ortho* positions, and proceeds to disubstitution at the *ortho* and *para* positions as the reaction continues. The absence of a trisubstituted product indicates that DIEA is not strong enough to facilitate triple substitution. This reaction also monitored without using any base, however no product was detected in absence of base.

**Scheme 3:**
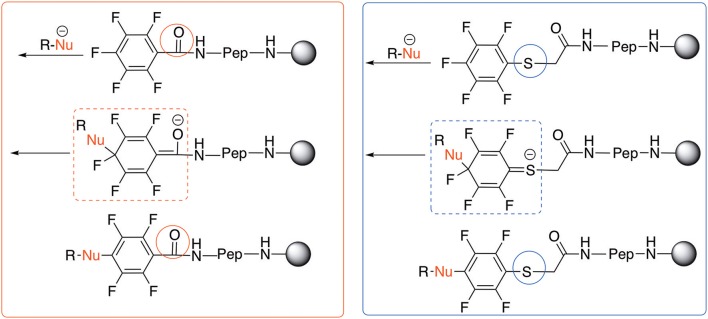
The reactivity of different nucleophiles.

Based on the report by Pentalute et al. having a S group can activate the *para* position and increase the reactivity of the fluorolinker for further substitution (Lühmann et al., 2016). However, based on our experiments, the same reactivity was observed with both **L**_1_ and **L**_2_, indicating that electron withdrawing groups such as the carbonyl group or electron donating groups such as thiols do not affect the reactivity or selectivity of the reaction. According to the mechanism (Scheme 3), the Meisenheimer complex can be stabilized by the delocation of the negative charge on either S in **L**_1_ or oxygen of the carbonyl group in **L**_2_. This explains why both **L**_1_ and **L**_2_ show the same reactivity toward different nucleophiles.

## Theoretical calculations

To elucidate the electronic effect on perfluorobenzene (Group I) and perfluorobiphenyl (Group II) conjugated on the tripeptide during the different nucleophilic substitution reactions, a density functional theory (DFT) geometry optimization calculation performed in the Gas phase was carried out. DFT in the Gaussian09 program package (Frisch et al., 2009) employing the B3LYP (Becke three parameters Lee–Yang–Parr) exchange correlation functional, which combines the hybrid exchange functional of Becke (1988) with the gradient-correlation functional of Lee et al. (1988) and the 6-31G(d) basis set was used. The frequency calculations afforded no negative Eigen values indicating stability of the molecule. After optimization of the molecules, their atomic charges were calculated using natural bond orbital (NBO) analysis, another efficient tool for studying hyperconjugative interactions, intermolecular charge transfer, and electron density transfer (EDT), which are fundamentally linked for calculations of atomic charges (Drissi et al., 2015; Md Abdur Rauf et al., 2015; El-Faham et al., 2016; Kouakou Nobel et al., 2017).

In the present work, NBO analysis was performed on all derivatives including one nucleophilic substitution on Group I and II, and comparison was made on the basis of the charge carried by the fluorine atom (Supplementary Data). Table 3 shows the electronic charges present on fluorine in all molecules. As seen from Table 3, the fluorine atom in Group I and II carries a negative charge due to high electronegativity of the fluorine atom and are quite similar upon comparison of Group I and II, which indicates identical reactivity toward nucleophilic substitution at the *para* position (as confirmed by ^19^F NMR).

**Table 3:** Charges carried by the fluorine atoms in molecules calculated using NBO calculations.

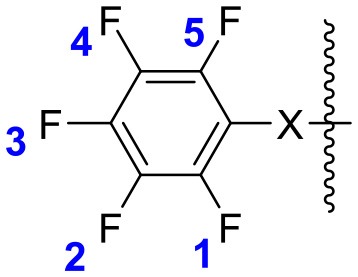							
		X = -CO-Gly-Phe-Leu-NH_**2**_, Group I X = -S-CH_**2**_-CO-Gly-Phe-Leu-NH_**2**_, Group II					
**Entry**		**1-F** **(1-C)**	**2-F** **(2-C)**	**3-F** **(3-C)**	**4-F** **(4-C)**	**5-F** **(5-C)**	**–** **(6-C)**
1	Group I	−0.317 (0.371)	−0.306 (0.297)	−0.301 (0.319)	−0.304 (0.299)	−0.304 (0.393)	– (−0.255)
2	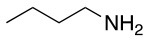	−0.322 (0.376)	−0.330 (0.312)	– (0.081)	−0.325 (0.308)	−0.309 (0.404)	– (−0.283)
3	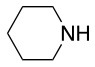	−0.323 (0.371)	−0.321 (0.337)	– (0.072)	−0.322 (0.323)	−0.310 (0.396)	– (−0.267)
4	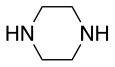	−0.323 (0.371)	−0.321 (0.337)	– (0.071)	−0.322 (0.324)	−0.310 (0.396)	– (−0.265)
5	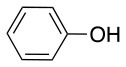	−0.319 (0.369)	−0.307 (0.333)	– (0.197)	−0.307 (0.323)	−0.307 (0.390)	– (−0.254)
6	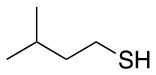	−0.320 (0.369)	−0.309 (0.363)	– (−0.318)	−0.316 (0.358)	−0.308 (0.389)	– (−0.241)
7	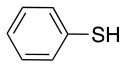	−0.320 (0.368)	−0.310 (0.359)	– (−0.321)	−0.308 (0.364)	−0.308 (0.389)	– (−0.241)
8	Group II	−0.314 (0.360)	−0.306 (0.298)	−0.302 (0.316)	−0.305 (0.299)	−0.305 (0.368)	– (−0.347)
9	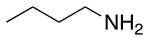	−0.319 (0.371)	−0.326 (0.306)	– (0.080)	−0.330 (0.314)	−0.311 (0.374)	– (−0.372)
10	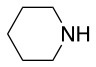	−0.320 (0.360)	−0.321 (0.338)	– (0.069)	−0.323 (0.324)	−0.311 (0.371)	– (−0.355)
11	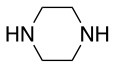	−0.320 (0.361)	−0.321 (0.339)	– (0.068)	−0.323 (0.324)	−0.311 (0.371)	– (−0.354)
12	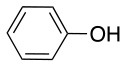	−0.316 (0.358)	−0.307 (0.334)	– (0.193)	−0.308 (0.326)	−0.308 (0.365)	– (−0.343)
13	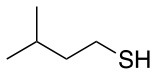	−0.318 (0.356)	−0.310 (0.364)	– (−0.322)	−0.316 (0.359)	−0.309 (0.363)	– (−0.332)
14	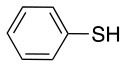	−0.318 (0.355)	−0.311 (0.361)	– (−0.323)	−0.310 (0.367)	−0.309 (0.363)	– (−0.331)

Due to the multiple substitution at the *ortho* and *para* positions in case of thiol, comparison of charge carried by fluorine after the first substitution was also studied (Table 3). To ease the explanation, the fluorine atom in the molecule was labeled as 1-F, 2-F, 3-F, 4-F, and 5-F. As explained by NMR studies, the substitution of 3-F by nucleophiles was observed which changed the atomic charges on remaining fluorine atoms in both cases. It was observed that after substitution by amines as nucleophile in the case of Group I and II, the charges carried by remaining fluorine atoms attain a more negative value i.e., ~-0.320 to −0.330 from that of −0.304 to −0.322, which may be the reason for making the next substitution difficult under the same reaction conditions leading to the formation of only mono-substituted product.

On the other hand, when phenol was used as a nucleophile, a similar increase of charge on fluorine was witnessed (as explained above), and hence explained the mono-substituted product. In the case of substitution by thiol at the *para* position, the charge carried by the rest of the fluorine atoms (# 6, 7, 13, and 14) is still comparable to that of Group I and II (# 1 and 8) especially for 1-F and 5-F, respectively, explaining the formation of the three substituted products (#6, 7, 13, and 14), irrespective of the nucleophile being aliphatic or aromatic as a similar conclusion was drawn in both cases.

## Conclusion

Using a solid phase approach, the reactivity and chemoselectivity of fluorine containing linkers for peptide conjugation was studied. Using DBU as a base and NMP as solvent, primary and secondary amines and phenols render the mono substituted product in the *para* position in both linkers. On the other hand, when thiols are used as the nucleophile, the tri-substituted product (at the *ortho* and *para* positions) takes place. Using thiols in the presence of a weaker base such as DIEA, a mixture of the mono- (*ortho* or *para*) and di-substituted (*ortho* and *para*) were obtained. The experimental data was corroborated further with theoretical calculations. Natural atomic charges were calculated and were also found to be consistent in explaining the experimental results. These two linkers could be used for the conjugation of amines and phenols and for the preparation of dendrimers in the case of thiols.

## Author contributions

All experiments, data analysis, discussion and the manuscript preparation were done by SA. The computational analysis and the discussion of computational data are done by AS. BdlT, DR, NK, and FA assisted with experiment design, manuscript preparation, and discussion. All authors have approved the submitted manuscript.

### Conflict of interest statement

The authors declare that the research was conducted in the absence of any commercial or financial relationships that could be construed as a potential conflict of interest.
