# Supplementary material for: Perfluorophenyl Derivatives as Unsymmetrical Linkers for Solid Phase Conjugation
Source: Front Chem. 2018 Nov 28;6:589. doi: 10.3389/fchem.2018.00589 (PMC6279949; doi:10.3389/fchem.2018.00589)
Supplement: Supplementary file 1 [file Data_Sheet_1.pdf]

Supplementary Material

Perfluorophenyl Derivatives as Unsymmetrical Linkers for Solid Phase Conjugation

Saba Alapour[1], Anamika Sharma[1,2], Beatriz G. de la Torre[2], Deresh Ramjugernath[3], Neil A. Koorbanally[1]*, and Fernando Albericio [1,3]*

*** Correspondence:**
Prof. Neil A. Koorbanally and Prof. Fernando Albericio
koorbanally@ukzn.ac.za and albericio@ukzn.ac.za

1. Materials, Methods and General Considerations

Material and Methods

Fmoc-protected amino acids and Rink amide resin were purchased from IRIS (Biotech). Oxyma Pure and DIC were bought from Luxembourg. HPLC–grade CH_3CN, MeOH and peptide synthesis-grade DMF, CH_2Cl_2, DIEA, TFA and all other reagents were purchased from Sigma-Aldrich (Germany). Analytical reversed- phase HPLC (instrument: LC-2030 C 3D [Promience-i]) was performed on a C18 column (2.1 × 100 mm, 5 µm, Shim-pack, Shimadzu, Kyoto, Japan) with a LC-MS 2020 system (Shimadzu, Kyoto, Japan). Solvents A and B were 0.01% (v/v) formic acid in double distilled water, and CH_3CN, respectively. LC-MS analysis was done using A:B gradient from 30 to 95% in 15 minutes and held at 95% A in B for 5 min, then column was flushed using 30% A in B for a period of five minutes), with UV detection at 220 nm. DMSO-d_6 was used as a solvent for sample preparation of NMR. ^{1}H NMR, ^{13}F NMR experiments were performed at ambient temperature on a AVANCE III 400 MHz NMR (Bruker, Germany). The ^{1}H NMR spectra were recorded with 400 MHz while ^{19} F NMR spectra were obtained with 376 MHz as the frequency, respectively.

General Procedure for peptide synthesis:

Peptides were synthesized on a 0.1 mmol scale on Rink amide resin using manual Fmoc-SPPS benchtop system using standard methods.

The procedure used for coupling cycle included a 20 min coupling with 0.3 mmol Fmoc protected amino acids, 0.3 mmol Oxyma Pure, 0.3 mmol DIC in 0.5 mL of DMF, 1 min wash with DCM and DMF. Deprotection with 20% (v/v) piperidine in DMF and 1min wash with DCM and DMF.

General Procedure for S_NAr reaction:

0.1 mmol of the resin was first suspended in 0.5 ml of solvent to swell for 30 s, then, base (10 eq., 1 mmol) and nucleophile (10 eq.,1 mmol) were added, and the reaction medium was mixed

using a shaker for the reported time. Then, the solvent was removed, and the resin washed with DMF (3 times) and DCM (3 times). The resin was placed in an Eppendorf tube and TFA was added to cleave the peptide from the resin. After 1 hrs, the mixture containing resin and TFA was filtered, and TFA present in the filtrate solution was evaporated. Then diethyl ether was added, and the peptide was precipitated out of the solution. The peptide was then collected from the ether using a centrifuge and dried. The crude product was used for characterisation without further purification or isolation.

LC of **L₁**

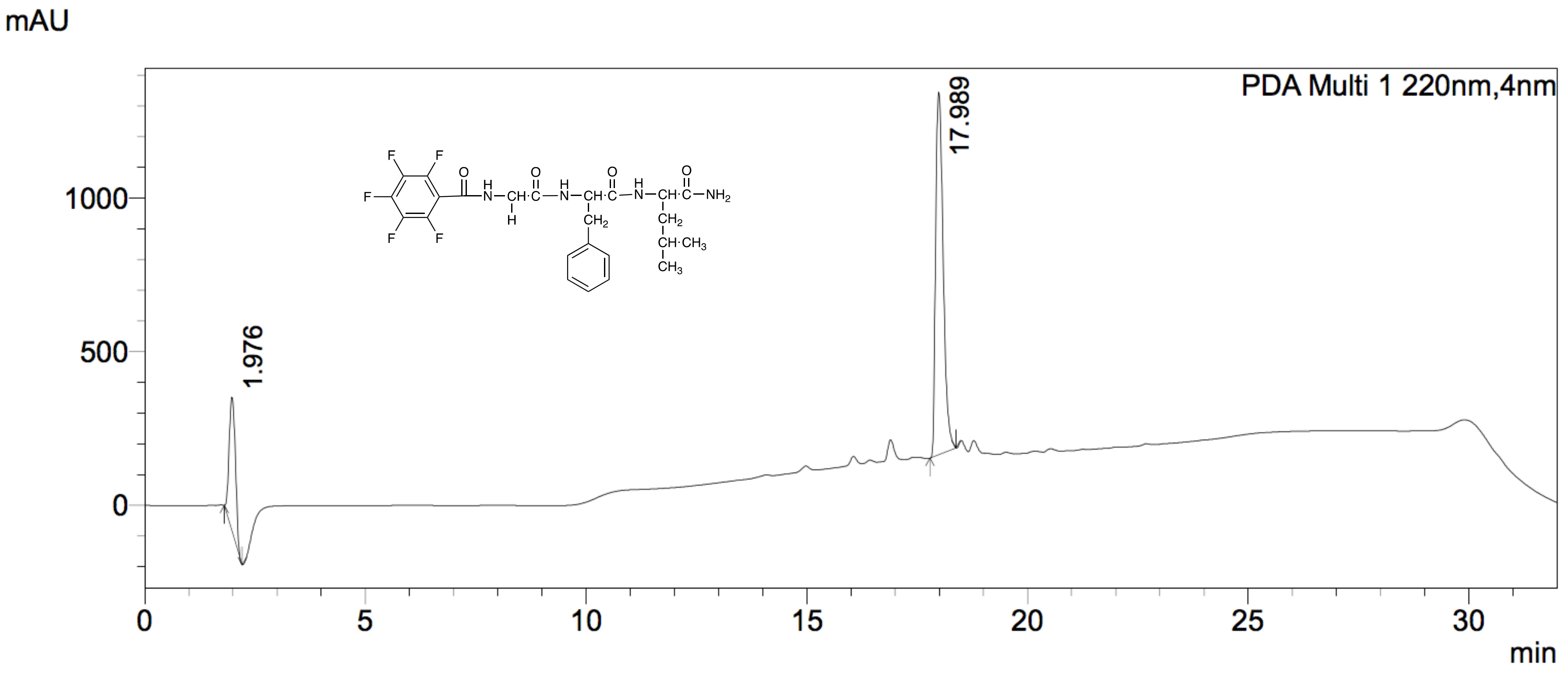

mAU
PDA Multi 1 220nm,4nm
1000
500
0
1.976
17.989
F
O
H
N
CH
C
NH2
CH2
CH·CH3
CH3
0
5
10
15
20
25
30
min

^{19}F NMR spectrum of **L1**

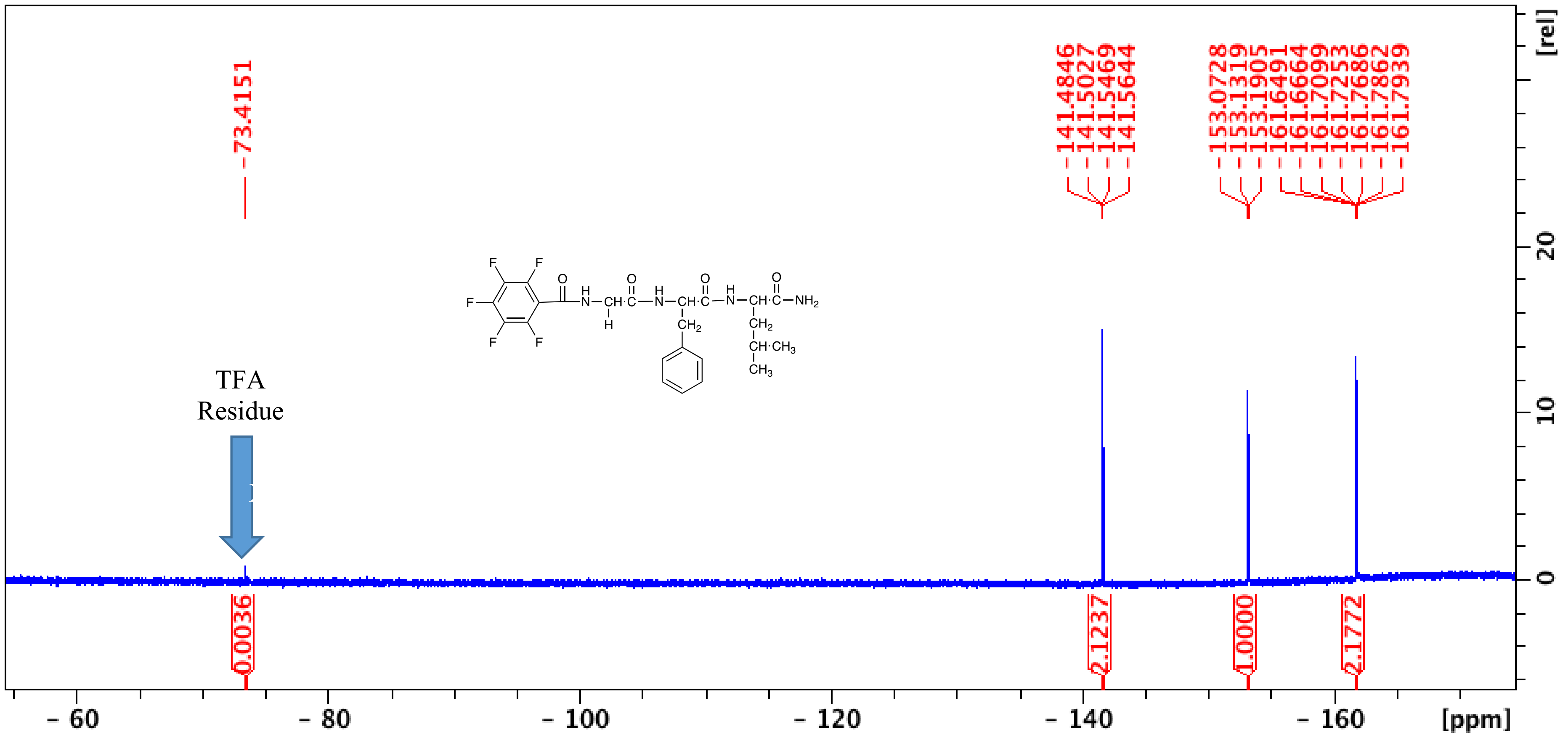

HRMS of **L**1

Single Mass Analysis

Tolerance = 5.0 PPM / DBE: min = -1.5, max = 50.0
Element prediction: Off
Number of isotope peaks used for i-FIT = 2

Monoisotopic Mass, Even Electron Ions
158 formula(e) evaluated with 1 results within limits (up to 20 closest results for each mass)
Elements Used:
C: 20-25 H: 20-25 N: 0-5 O: 0-5 F: 0-5 Na: 1-1
SFT 60 (1.990) Cm (1:61)
TOF MS ES+

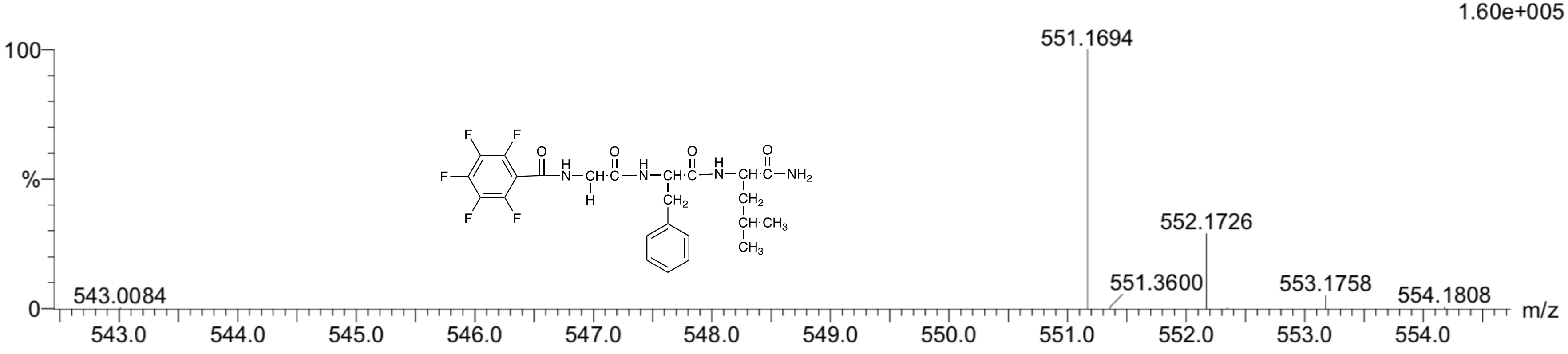

Minimum:				-1.5			
Maximum:		5.0	5.0	50.0			
Mass	Calc. Mass	mDa	PPM	DBE	i-FIT	i-FIT (Norm)	Formula
551.1694	551.1694	0.0	0.0	11.5	34.7	0.0	C24 H25 N4 O4 F5 Na

LC of **L2**

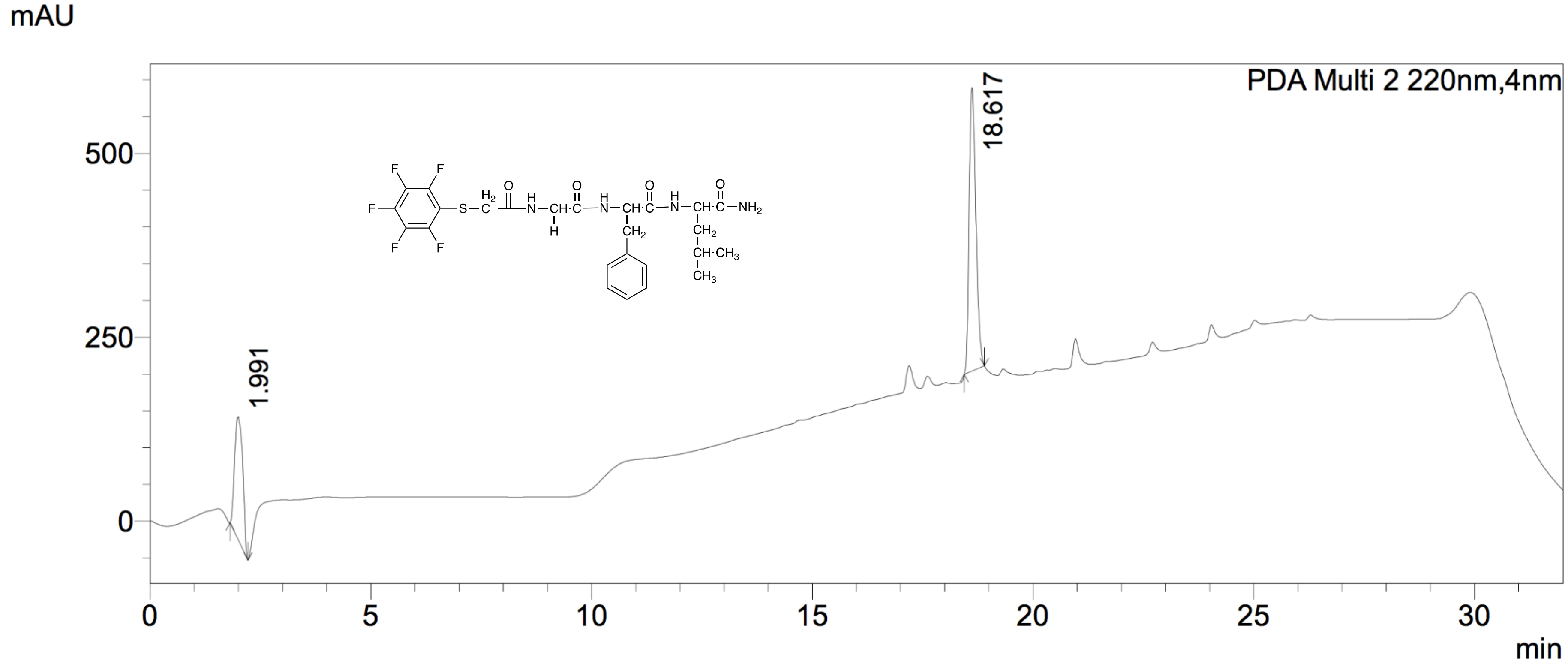

mAU
PDA Multi 2 220nm,4nm
500
250
0
1.991
18.617
0
5
10
15
20
25
30
min

^{19}F NMR spectrum of **L2**

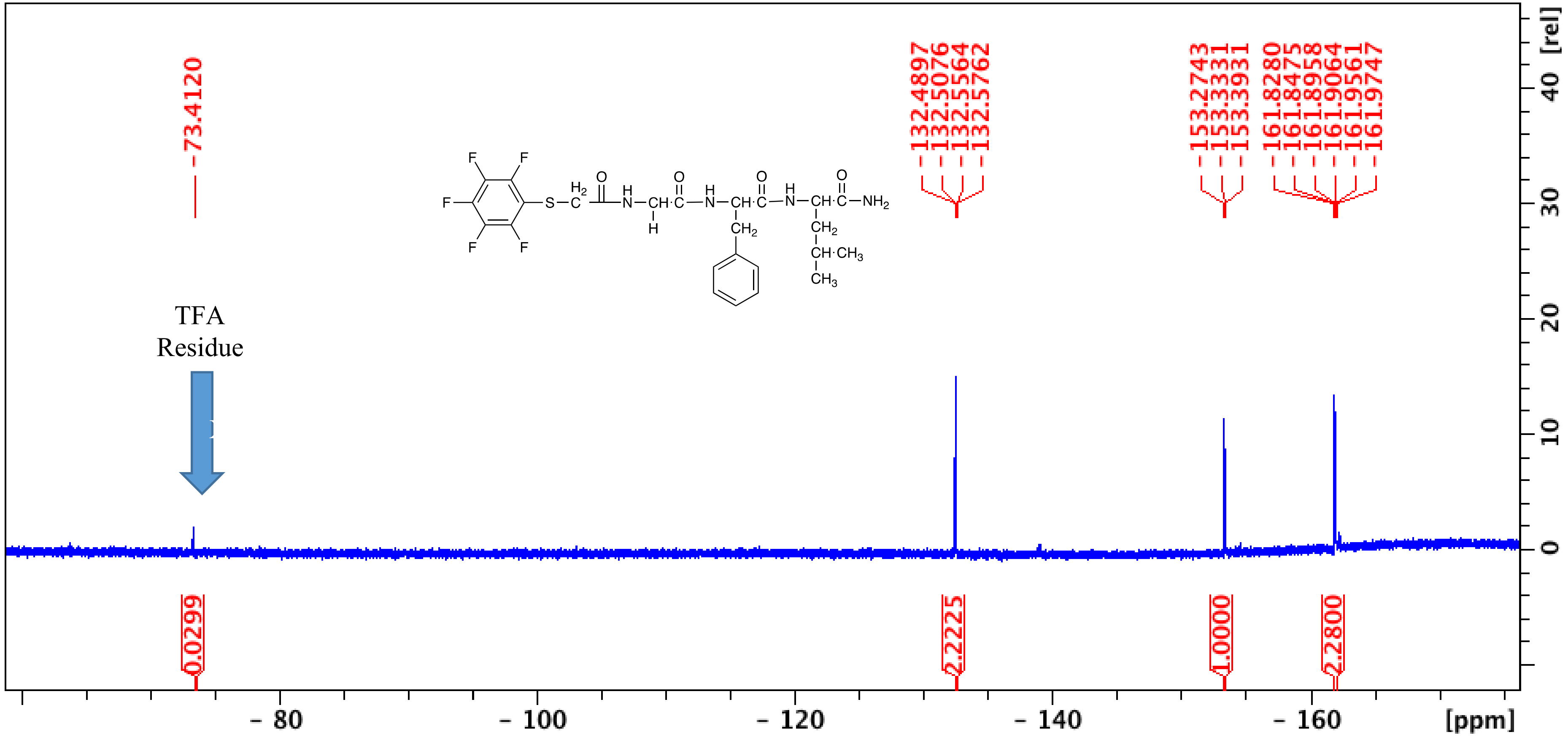

HRMS of **L_2**

Single Mass Analysis

Tolerance = 5.0 PPM / DBE: min = -1.5, max = 50.0
Element prediction: Off
Number of isotope peaks used for i-FIT = 2

Monoisotopic Mass, Even Electron Ions
371 formula(e) evaluated with 1 results within limits (up to 20 closest results for each mass)
Elements Used:
C: 20-25 H: 25-30 N: 0-5 O: 0-5 F: 0-5 Na: 1-1 S: 0-1
STS 9 (0.270) Cm (1:61)
TOF MS ES+

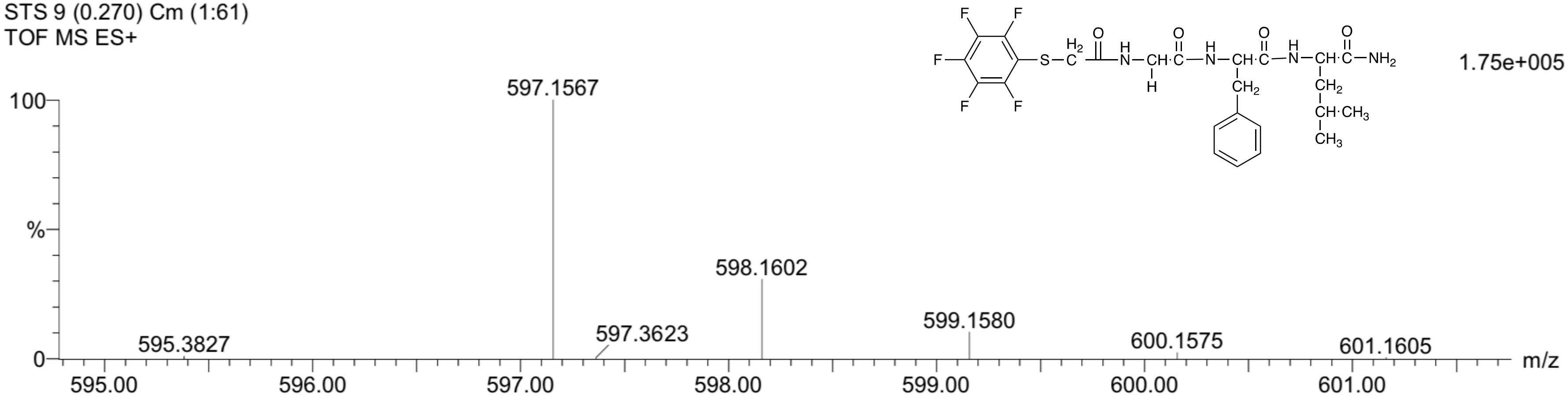

Minimum:				-1.5
Maximum:		5.0	5.0	50.0

Mass	Calc. Mass	mDa	PPM	DBE	i-FIT	i-FIT (Norm)	Formula
597.1567	597.1571	-0.4	-0.7	11.5	21.7	0.0	C25 H27 N4 O4 F5 Na S

LC of Entry **1,** Table **2**.

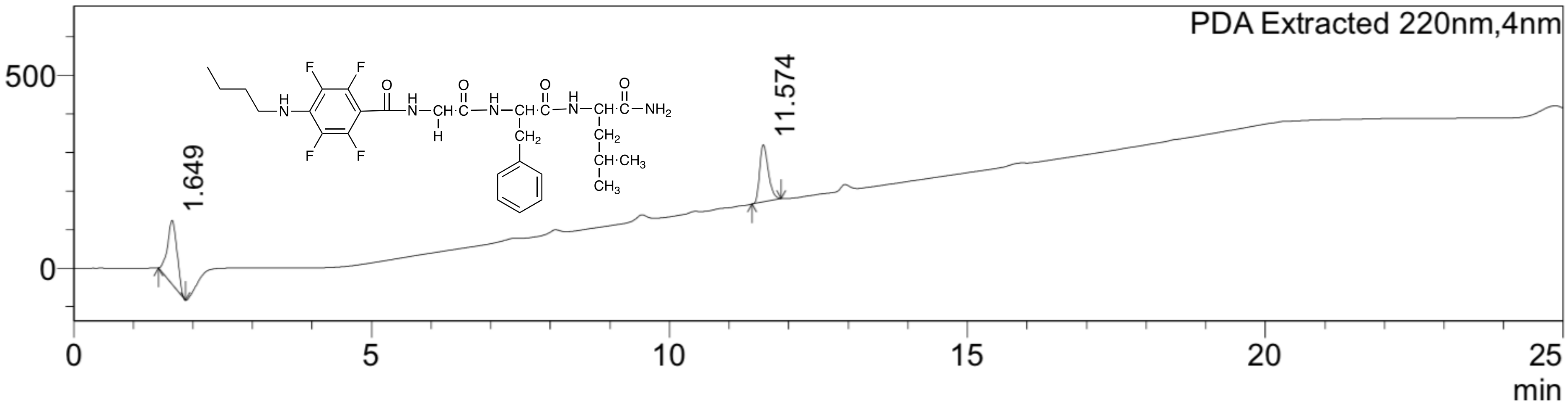

^{19}F NMR spectrum of Entry **1,** Table **2**.

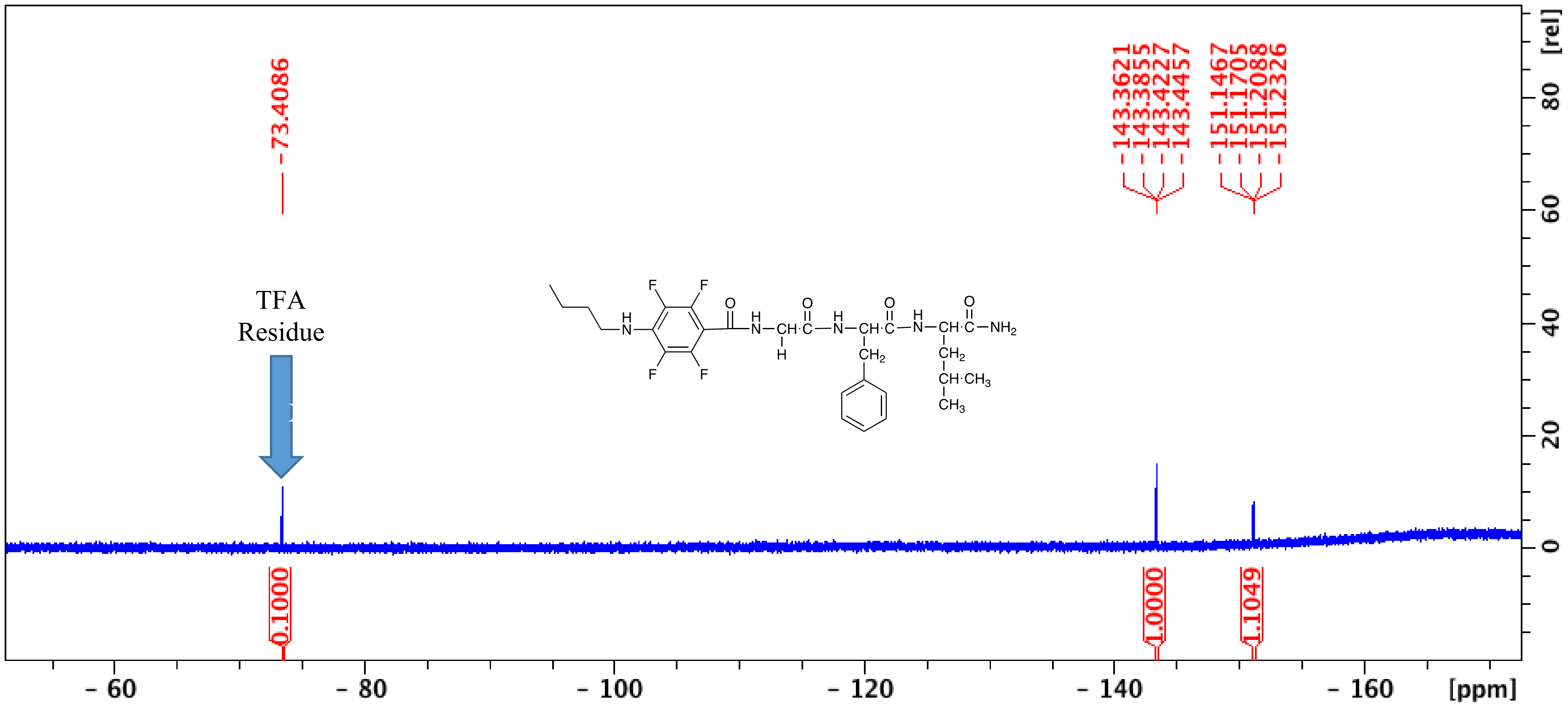

HRMS of Entry **1,** Table **2**.

Single Mass Analysis

Tolerance = 5.0 PPM / DBE: min = -1.5, max = 50.0
Element prediction: Off
Number of isotope peaks used for i-FIT = 2

Monoisotopic Mass, Even Electron Ions
123 formula(e) evaluated with 1 results within limits (up to 20 closest results for each mass)
Elements Used:
C: 25-30 H: 30-35 N: 0-5 O: 0-5 F: 0-5 Na: 1-1
SF1 51 (1.686) Cm (1:61)
TOF MS ES+

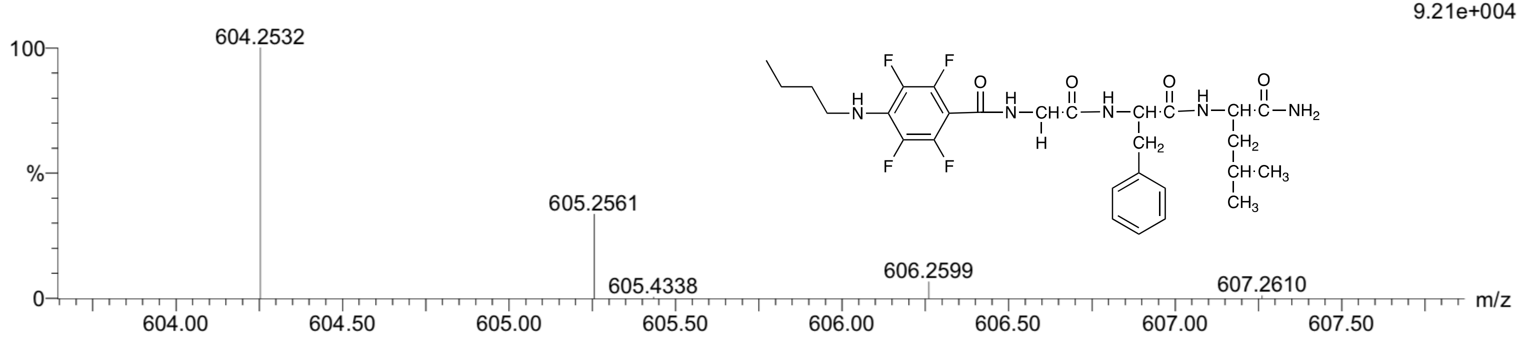

Minimum:				-1.5			
Maximum:		5.0	5.0	50.0			

Mass	Calc. Mass	mDa	PPM	DBE	i-FIT	i-FIT (Norm)	Formula
604.2532	604.2523	0.9	1.5	11.5	21.0	0.0	C28 H35 N5 O4 F4 Na

LC of Entry **2,** Table **2**.

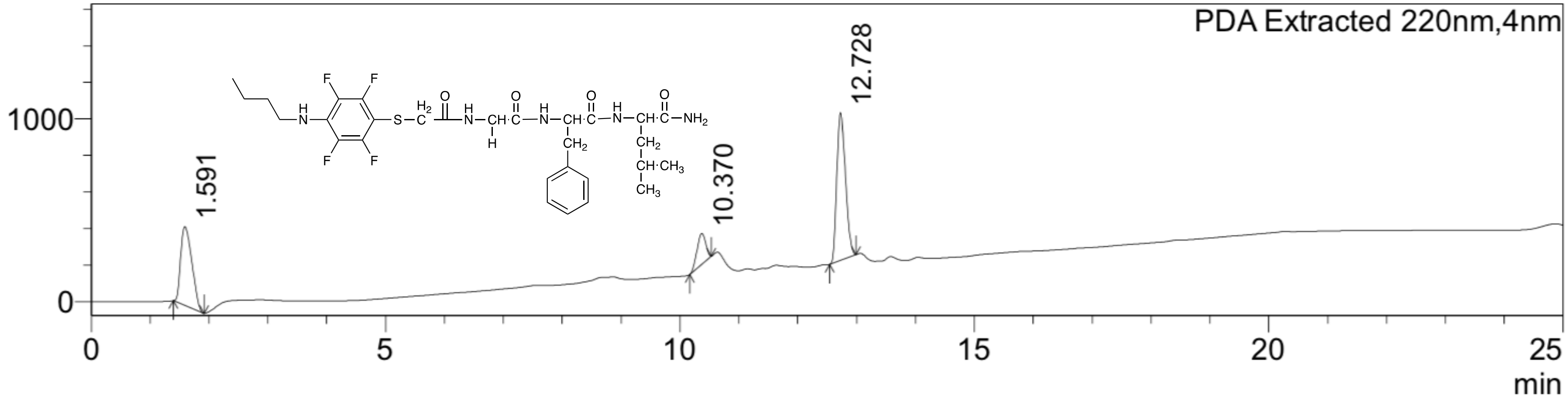

MS Chromatogram

^{19}F NMR spectrum of Entry **2,** Table **2**

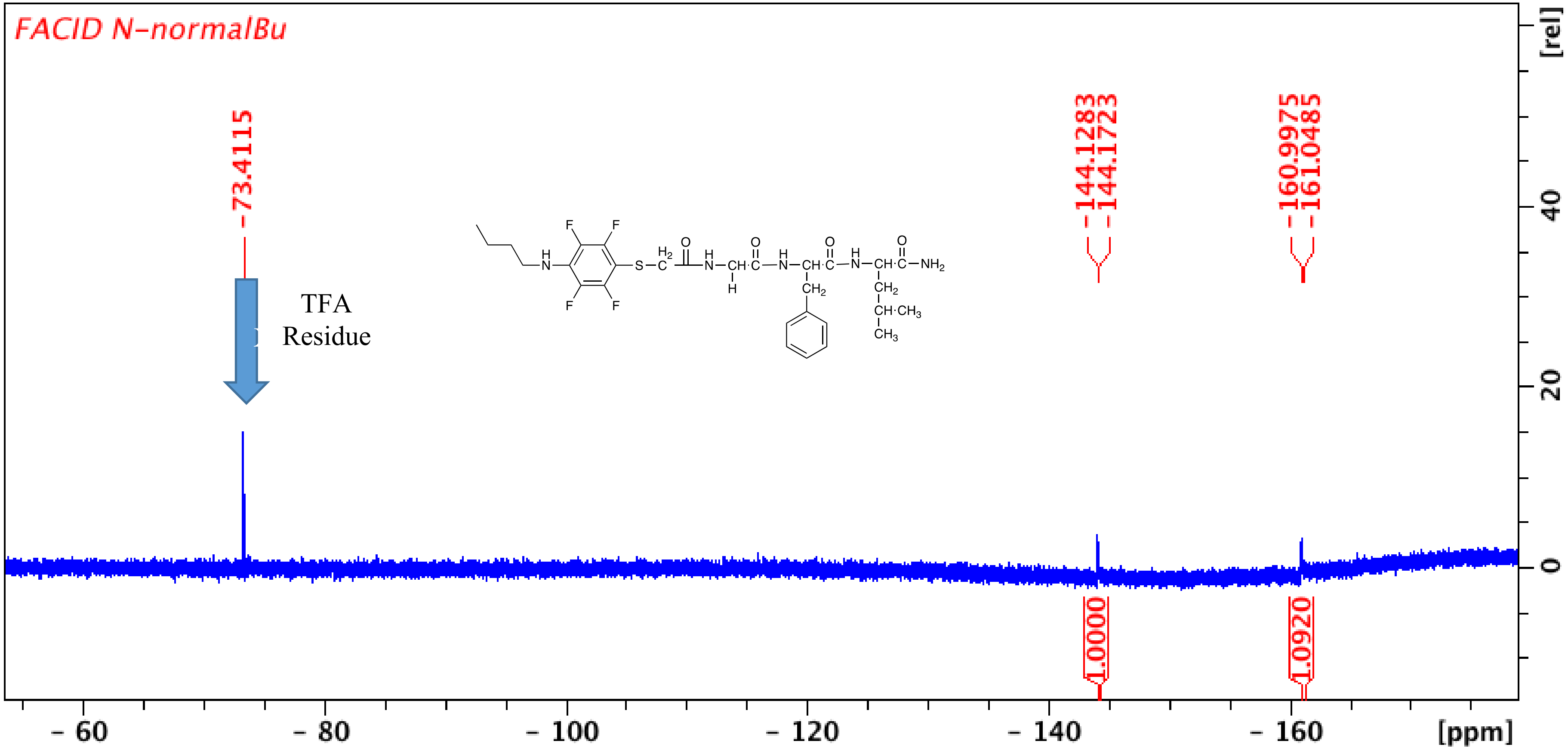

HRMS of Entry **2,** Table **2**

Single Mass Analysis

Tolerance = 5.0 PPM / DBE: min = -1.5, max = 50.0
Element prediction: Off
Number of isotope peaks used for i-FIT = 2

Monoisotopic Mass, Even Electron Ions
279 formula(e) evaluated with 1 results within limits (up to 20 closest results for each mass)
Elements Used:
C: 25-30 H: 35-40 N: 0-5 O: 0-5 F: 0-4 Na: 1-1 S: 0-1

SS 1 44 (1.451) Cm (1:61)
TOF MS ES+

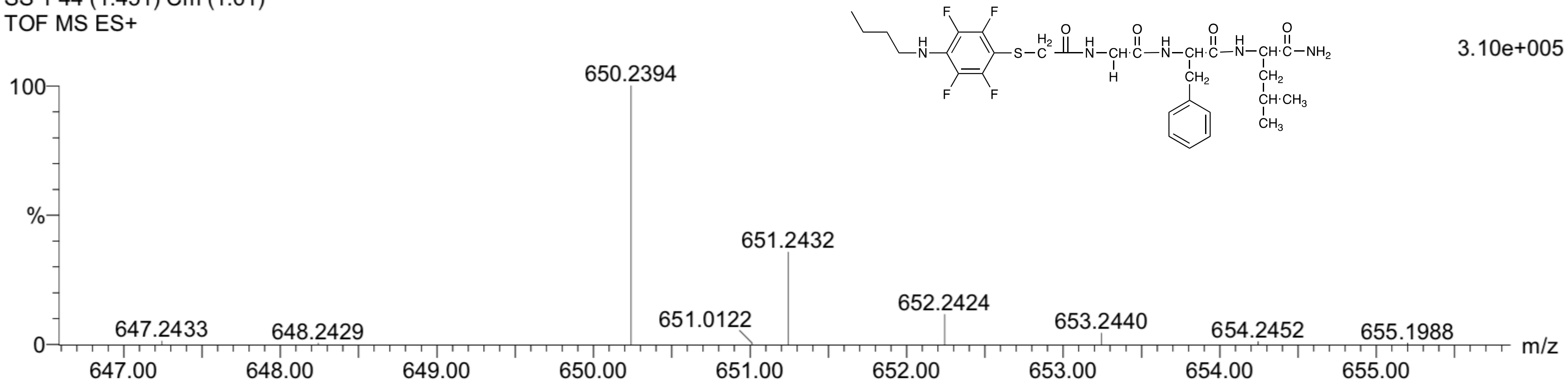

Minimum:				-1.5			
Maximum:		5.0	5.0	50.0			
Mass	Calc. Mass	mDa	PPM	DBE	i-FIT	i-FIT (Norm)	Formula
650.2394	650.2400	-0.6	-0.9	11.5	23.9	0.0	C29 H37 N5 O4 F4 Na S

LC of Entry **3,** Table **2**

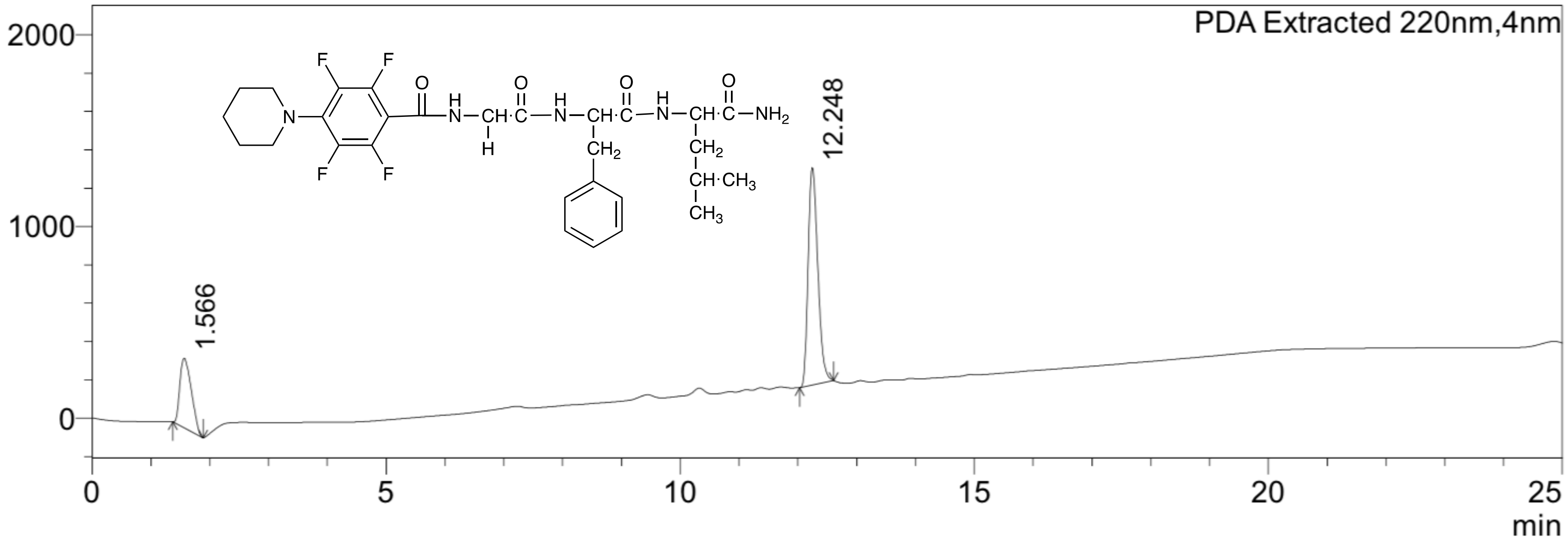

^{19}F NMR spectrum Entry **3,** Table **2**

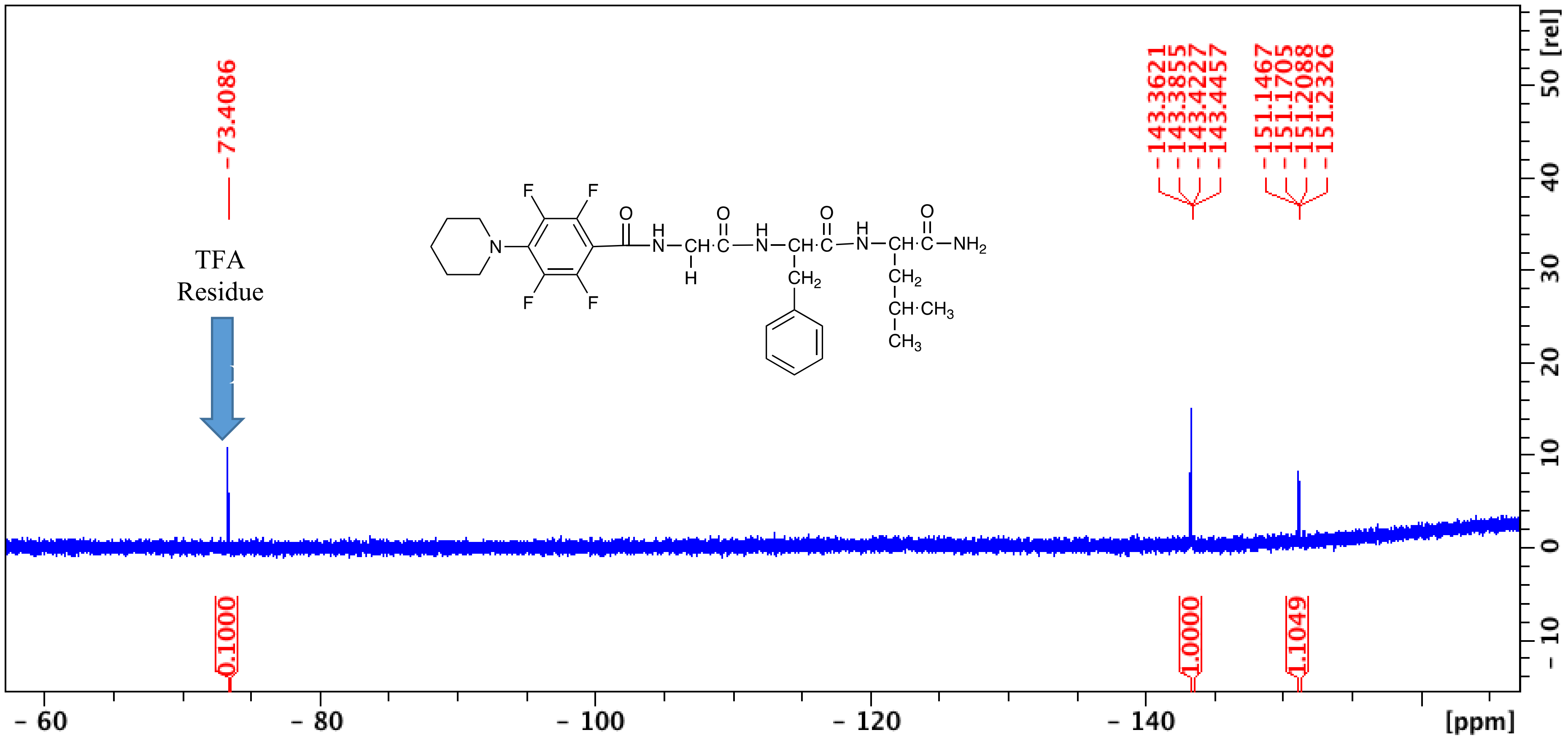

HRMS o Entry **3,** Table **2**

Single Mass Analysis

Tolerance = 5.0 PPM / DBE: min = -1.5, max = 50.0
Element prediction: Off
Number of isotope peaks used for i-FIT = 2

Monoisotopic Mass, Even Electron Ions
100 formula(e) evaluated with 1 results within limits (up to 20 closest results for each mass)
Elements Used:
C: 25-30 H: 30-35 N: 0-6 O: 1-4 F: 0-5 Na: 1-1
SF 5 42 (1.383) Cm (1:61)
TOF MS ES+

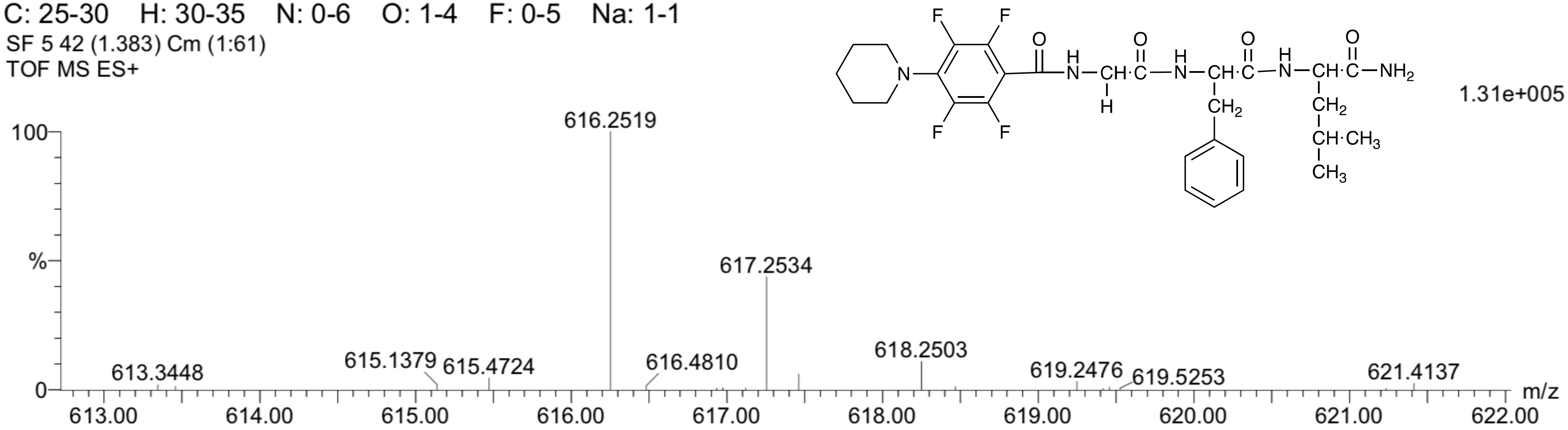

		mDa	PPM	DBE			
Minimum:				-1.5			
Maximum:		5.0	5.0	50.0			
Mass	Calc. Mass	mDa	PPM	DBE	i-FIT	i-FIT (Norm)	Formula
616.2519	616.2523	-0.4	-0.6	12.5	91.8	0.0	C29 H35 N5 O4 F4 Na

LC of Entry **4,** Table **2**

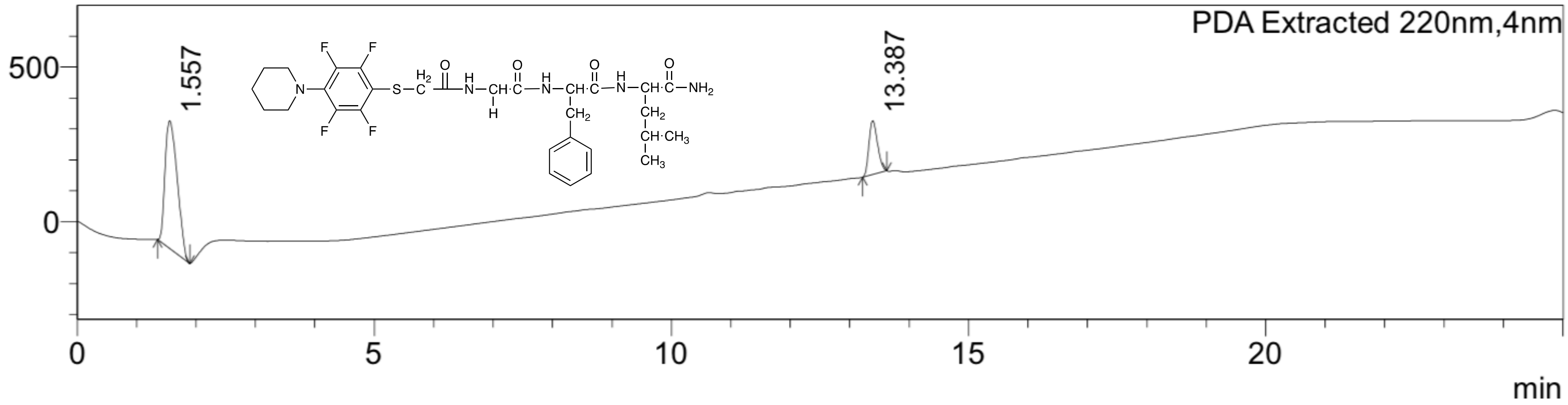

^{19}F NMR spectrum of Entry **4,** Table **2**

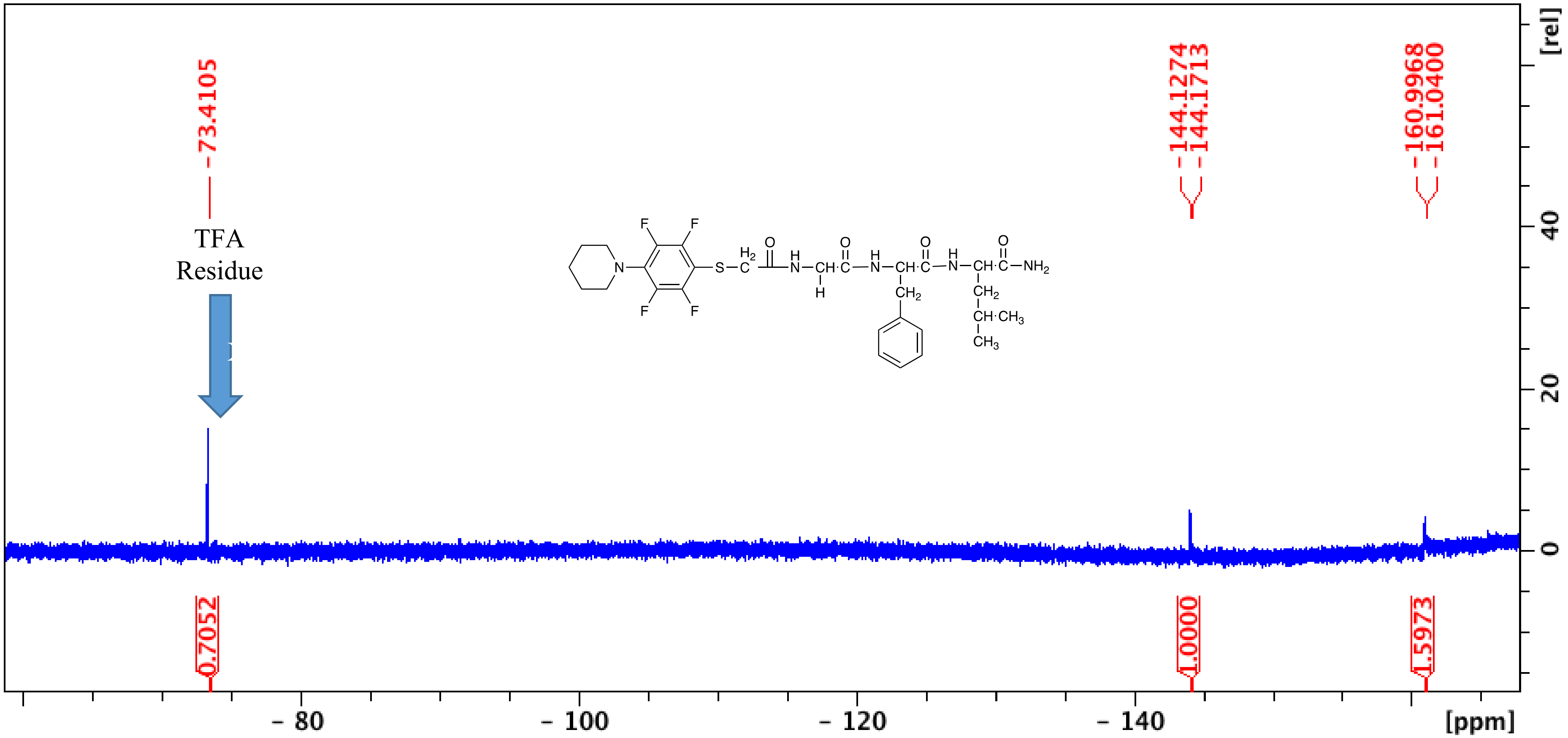

HRMS of Entry **4,** Table **2**

Single Mass Analysis

Tolerance = 5.0 PPM / DBE: min = -1.5, max = 50.0
Element prediction: Off
Number of isotope peaks used for i-FIT = 2

Monoisotopic Mass, Even Electron Ions
301 formula(e) evaluated with 1 results within limits (up to 20 closest results for each mass)
Elements Used:
C: 25-30 H: 35-40 N: 0-5 O: 0-5 F: 0-4 Na: 1-1 S: 0-1
SS 4 26 (0.843) Cm (1:61)
TOF MS ES+

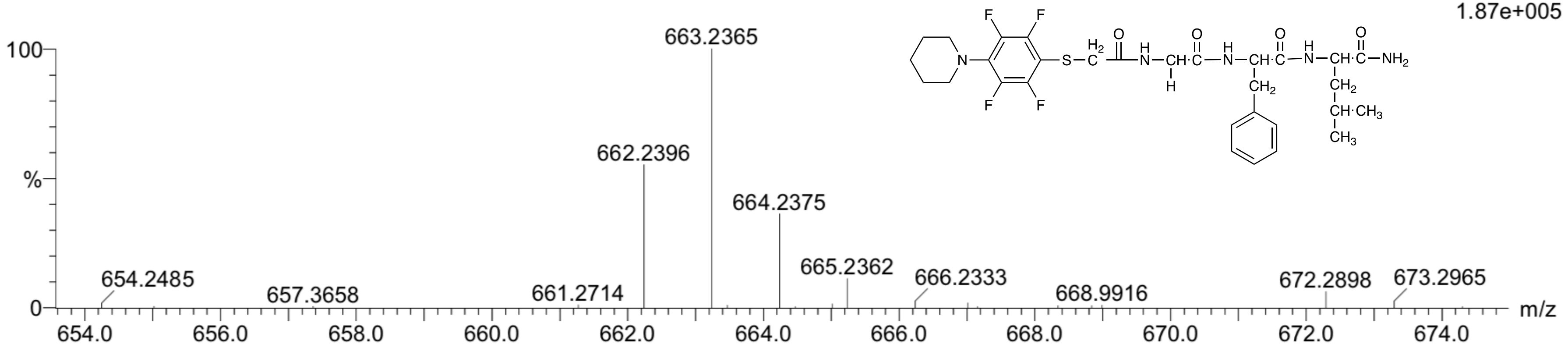

Minimum:				-1.5			
Maximum:		5.0	5.0	50.0			
Mass	Calc. Mass	mDa	PPM	DBE	i-FIT	i-FIT (Norm)	Formula
662.2396	662.2400	-0.4	-0.6	12.5	45.8	0.0	C30 H37 N5 O4 F4 Na S

LC of Entry **5,** Table **2**

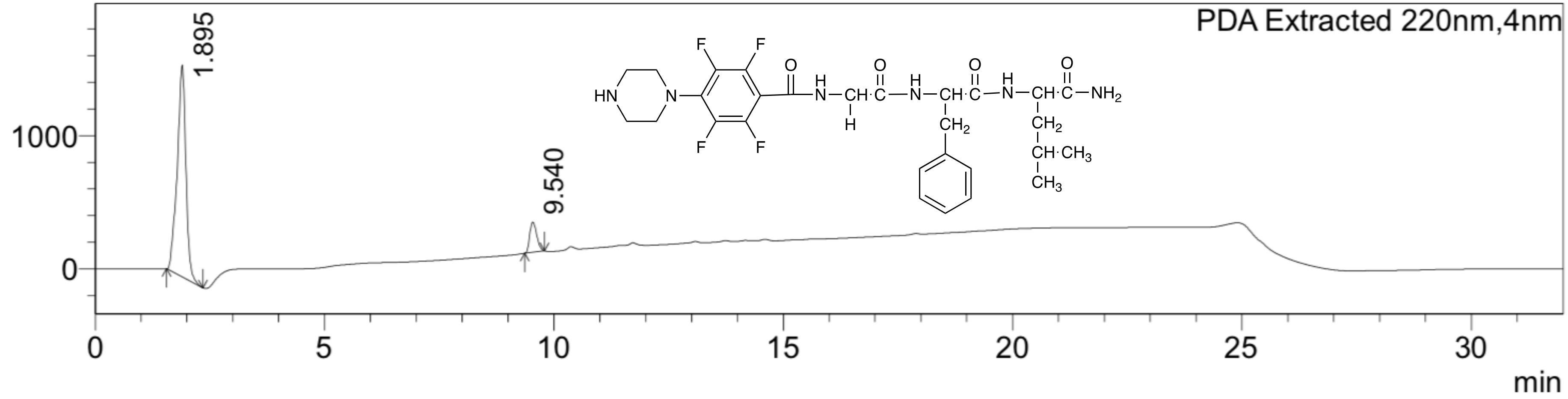

^{19}F NMR spectrum of Entry **5,** Table **2**

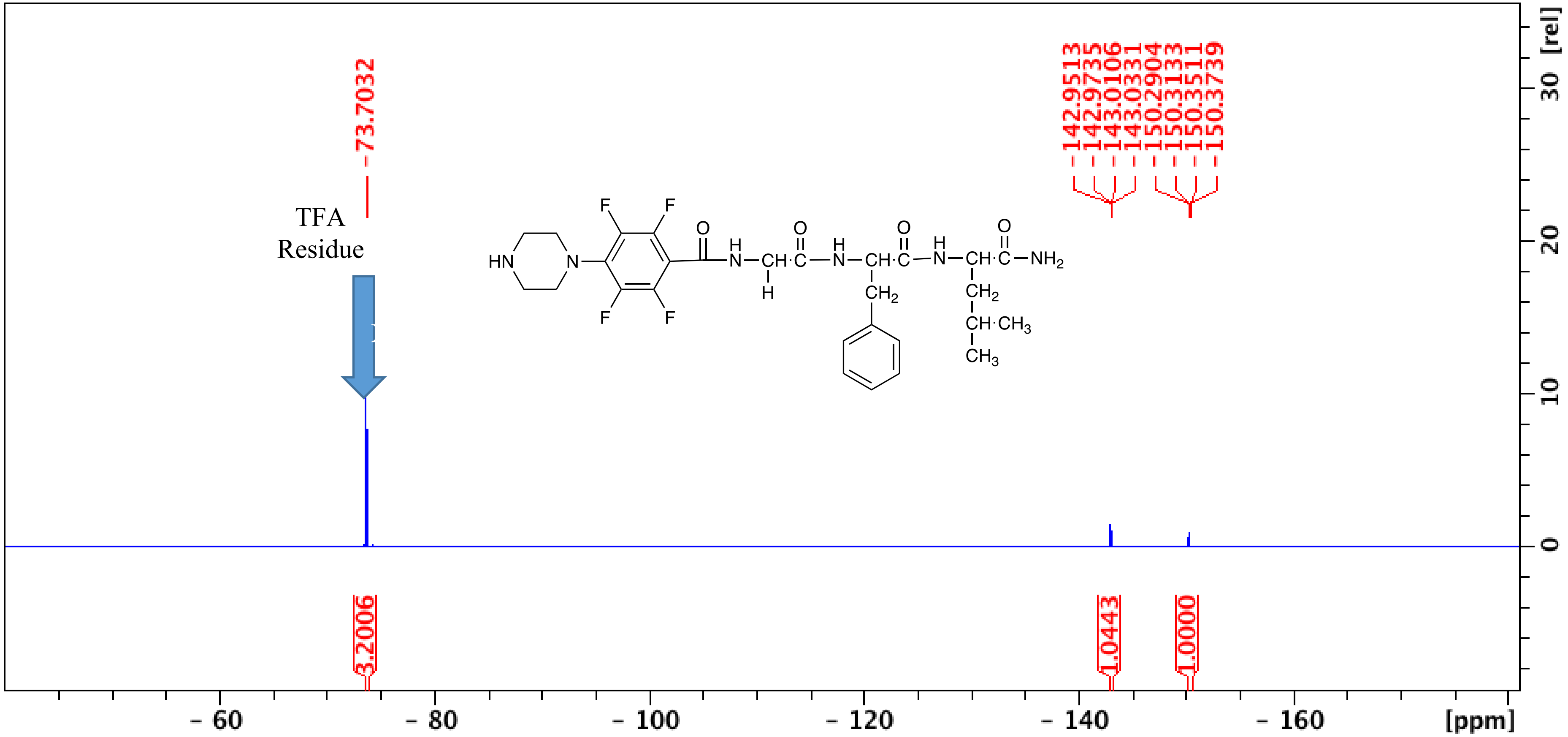

HRMS of Entry **5,** Table **2**

Single Mass Analysis

Tolerance = 5.0 PPM / DBE: min = -1.5, max = 50.0
Element prediction: Off
Number of isotope peaks used for i-FIT = 2

Monoisotopic Mass, Even Electron Ions
100 formula(e) evaluated with 1 results within limits (up to 20 closest results for each mass)
Elements Used:
C: 25-30 H: 30-35 N: 0-6 O: 1-4 F: 0-5 Na: 1-1
SF 4 47 (1.552) Cm (1:61)
TOF MS ES+

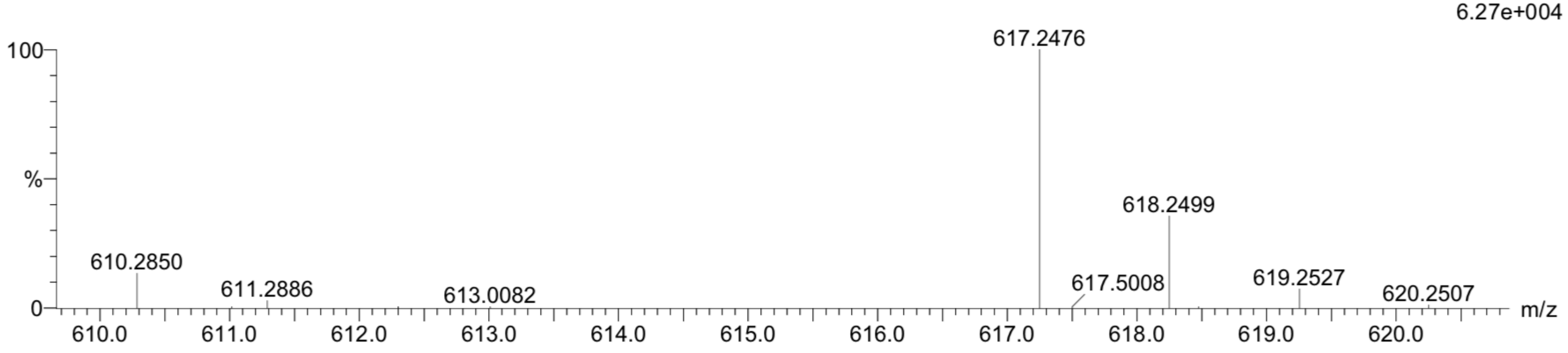

Minimum:				-1.5			
Maximum:		5.0	5.0	50.0			
Mass	Calc. Mass	mDa	PPM	DBE	i-FIT	i-FIT (Norm)	Formula
617.2476	617.2475	0.1	0.2	12.5	34.3	0.0	C28 H34 N6 O4 F4 Na

LC of Entry **6,** Table **2**

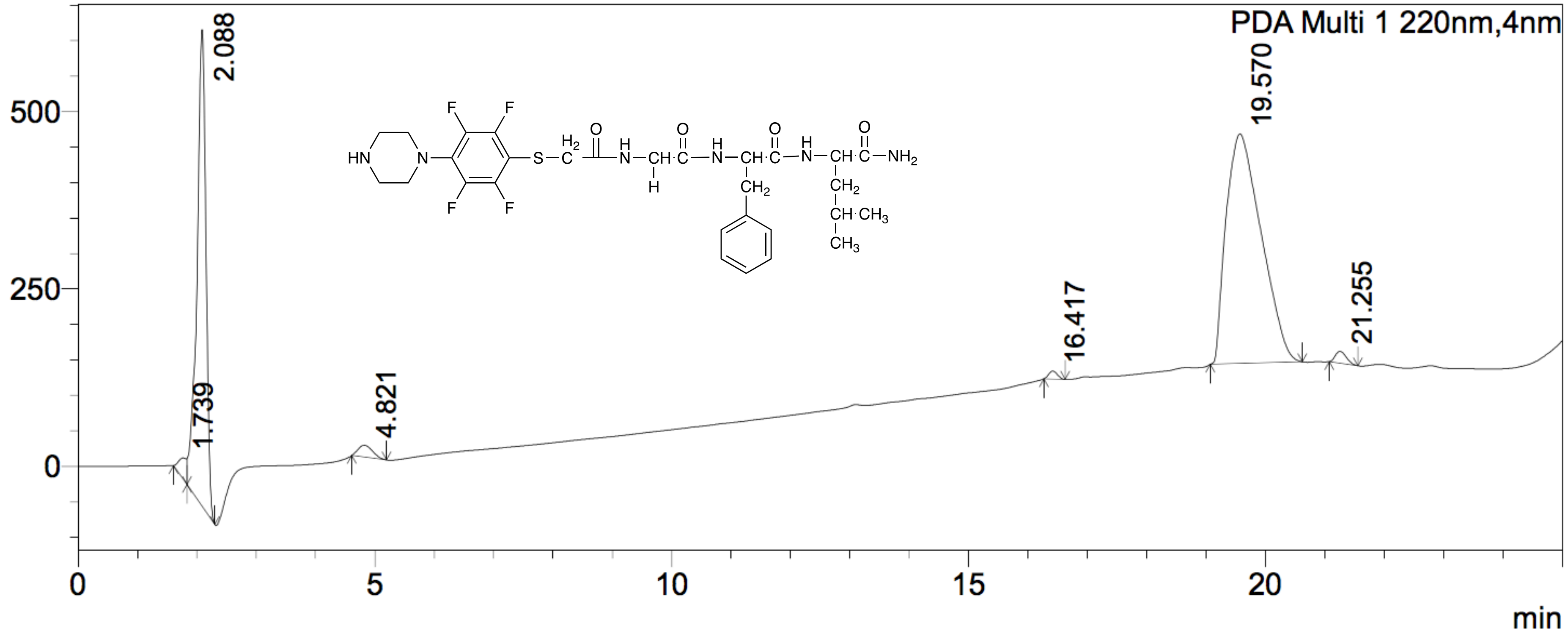

^{19}F NMR spectrum of Entry **6,** Table **2**

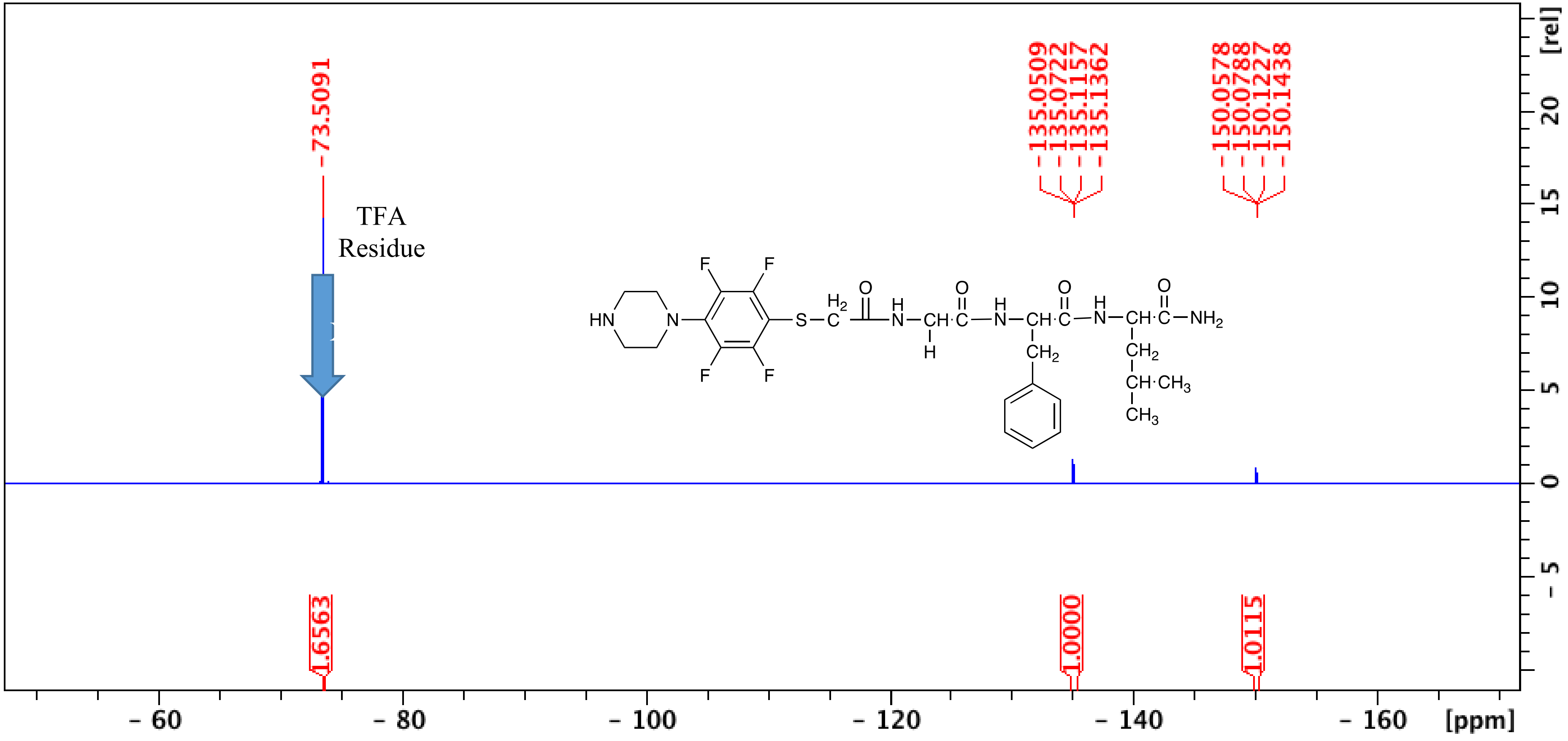

HRMS of Entry **6,** Table **2**

Single Mass Analysis

Tolerance = 5.0 PPM / DBE: min = -1.5, max = 50.0
Element prediction: Off
Number of isotope peaks used for i-FIT = 2

Monoisotopic Mass, Even Electron Ions
202 formula(e) evaluated with 1 results within limits (up to 20 closest results for each mass)
Elements Used:
C: 25-30 H: 35-40 N: 0-6 O: 1-5 F: 1-4 S: 0-1
SS 3 3 (0.068) Cm (1:61)
TOF MS ES+

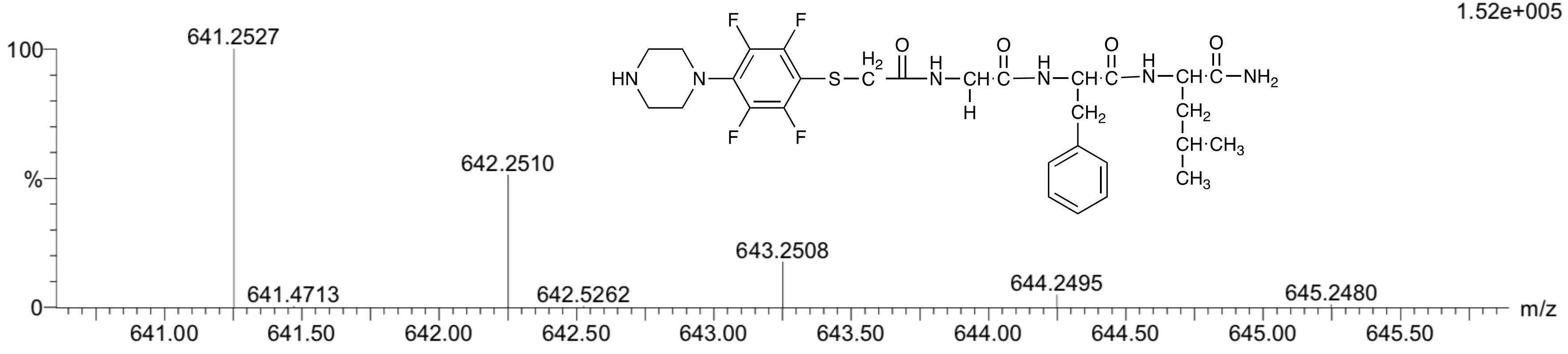

Minimum:				-1.5			
Maximum:		5.0	5.0	50.0			
Mass	Calc. Mass	mDa	PPM	DBE	i-FIT	i-FIT (Norm)	Formula
641.2527	641.2533	-0.6	-0.9	12.5	50.3	0.0	C29 H37 N6 O4 F4 S

LC of Entry **7,** Table **2**

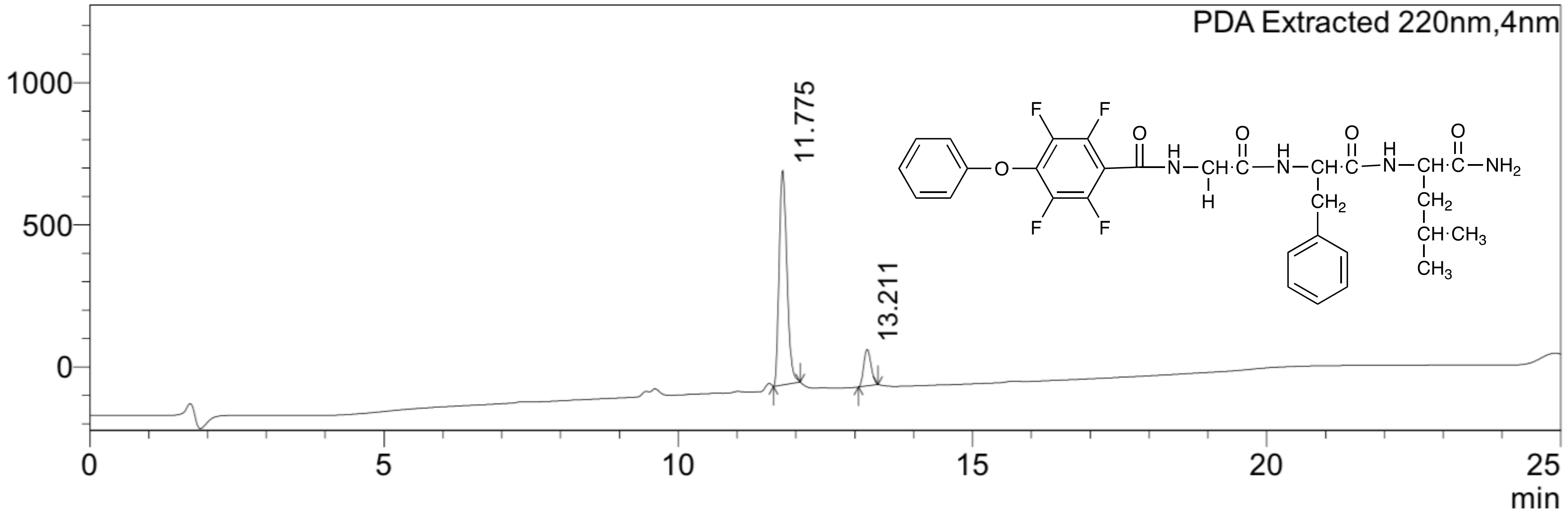
PDA Extracted 220nm,4nm
1000
500
0
11.775
13.211
0
5
10
15
20
25
min

^{19}F NMR spectrum LC of Entry **7,** Table **2**

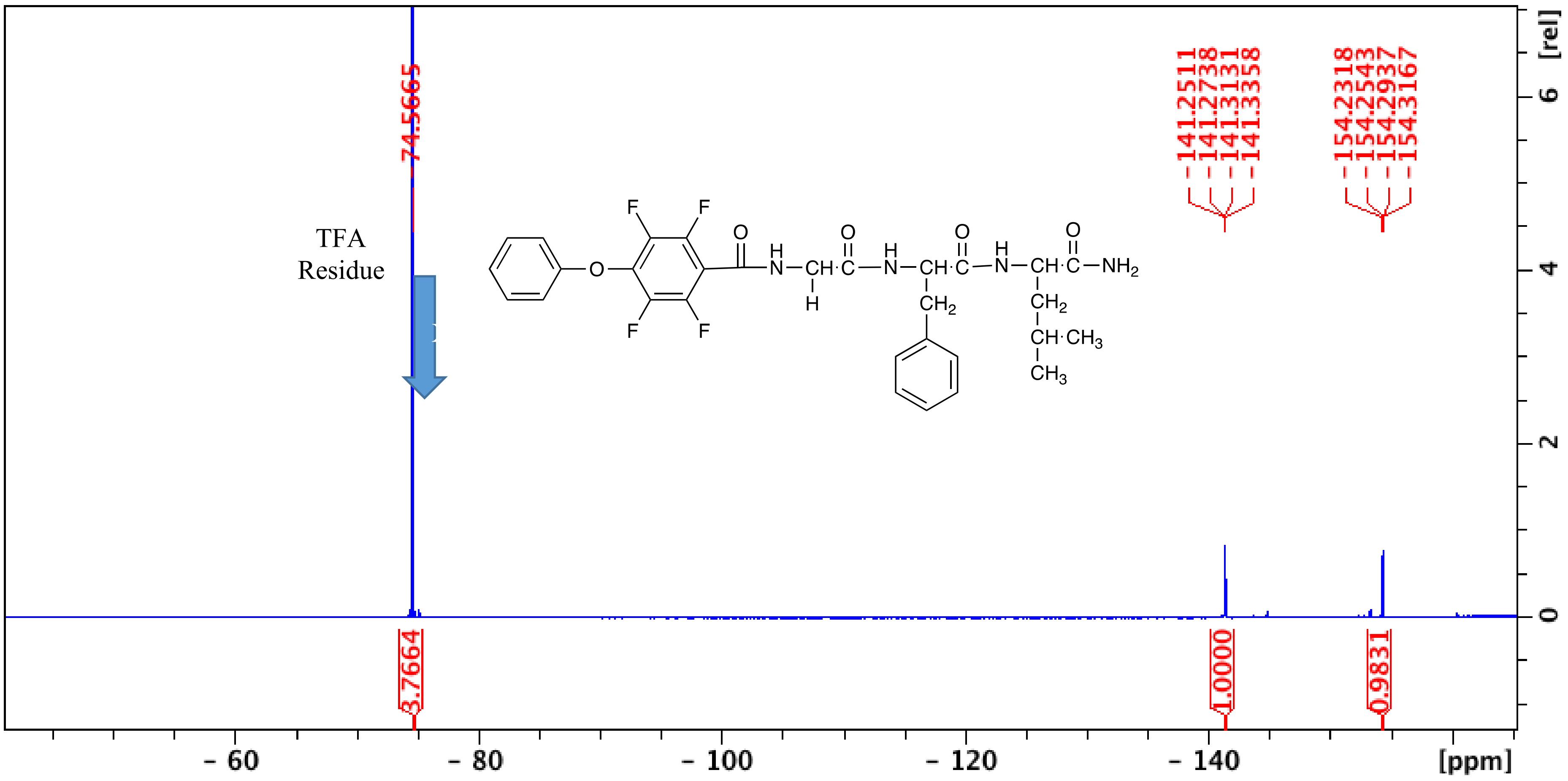

HRMS of LC of Entry **7,** Table **2**

Single Mass Analysis

Tolerance = 5.0 PPM / DBE: min = -1.5, max = 50.0
Element prediction: Off
Number of isotope peaks used for i-FIT = 2

Monoisotopic Mass, Even Electron Ions
156 formula(e) evaluated with 1 results within limits (up to 20 closest results for each mass)
Elements Used:
C: 25-30 H: 25-30 N: 0-5 O: 0-5 F: 0-5 Na: 1-1

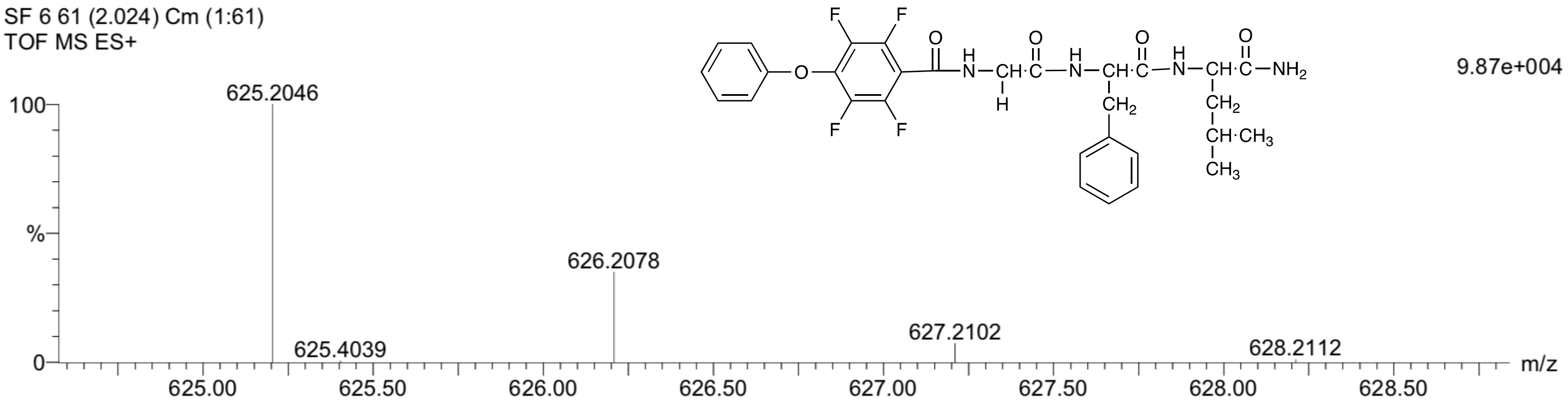

	Mass	Calc. Mass	mDa	PPM	DBE	i-FIT	i-FIT (Norm)	Formula
Minimum:					-1.5			
Maximum:			5.0	5.0	50.0			
	625.2046	625.2050	-0.4	-0.6	15.5	22.5	0.0	C30 H30 N4 O5 F4 Na

LC of Entry **8,** Table **2**

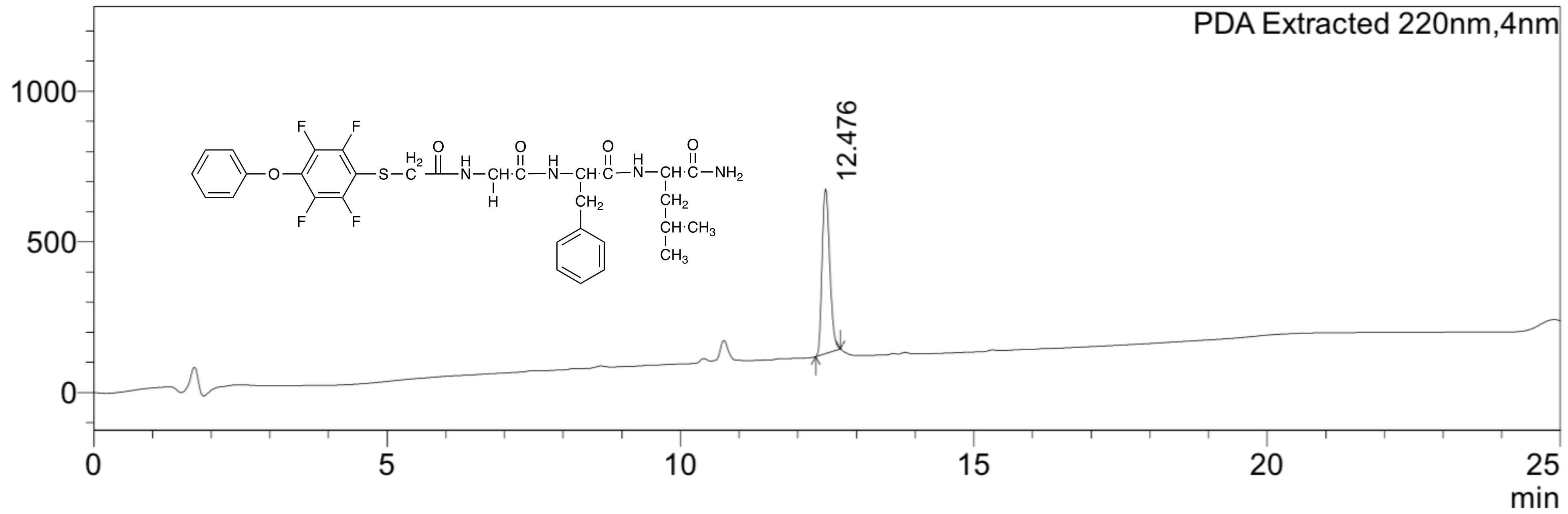

^{19}F NMR spectrum of Entry **8,** Table **2**

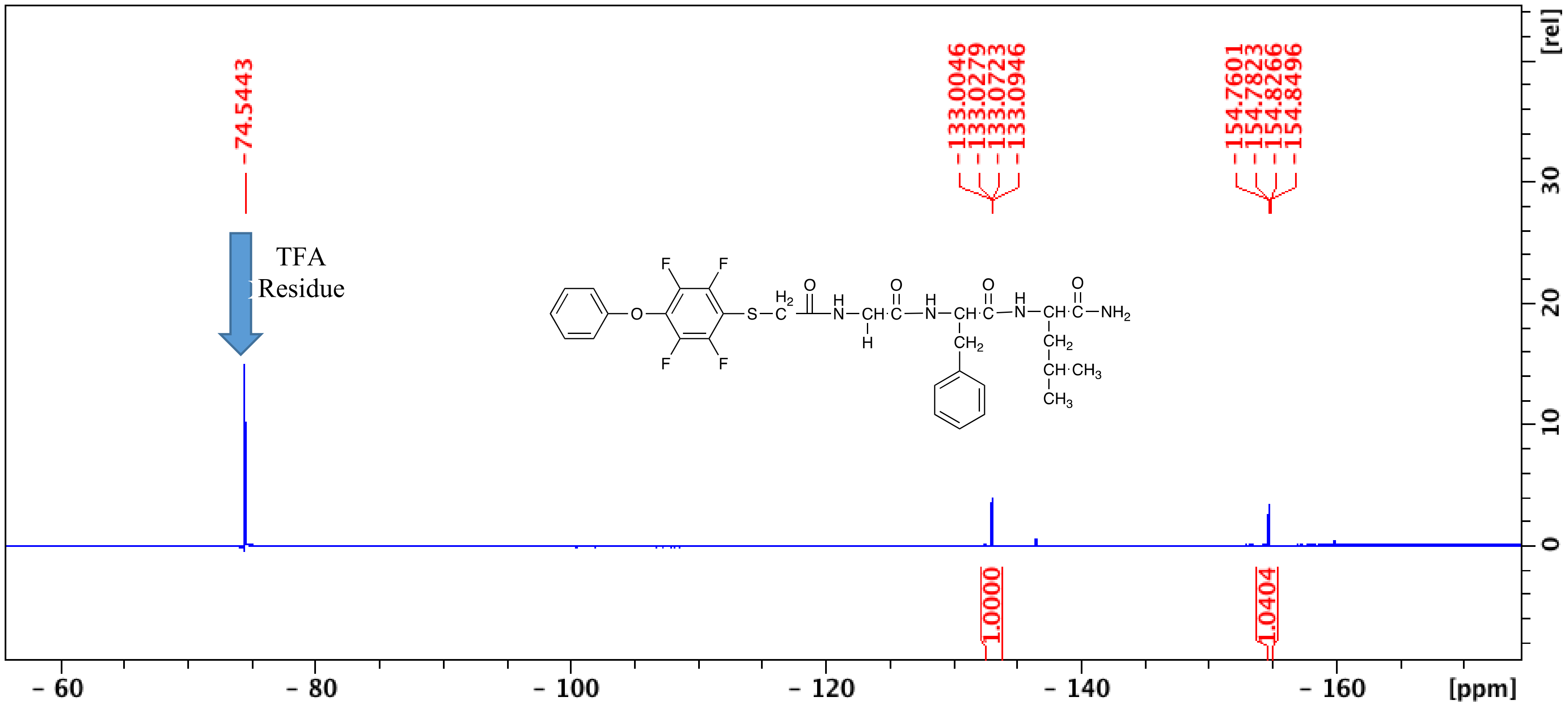

HRMS of Entry **8,** Table **2**

Single Mass Analysis

Tolerance = 5.0 PPM / DBE: min = -1.5, max = 50.0
Element prediction: Off
Number of isotope peaks used for i-FIT = 2

Monoisotopic Mass, Even Electron Ions
301 formula(e) evaluated with 1 results within limits (up to 20 closest results for each mass)
Elements Used:
C: 25-31 H: 30-35 N: 0-5 O: 0-5 F: 0-4 Na: 1-1 S: 0-1
SS 5 17 (0.540) Cm (1:61)
TOF MS ES+

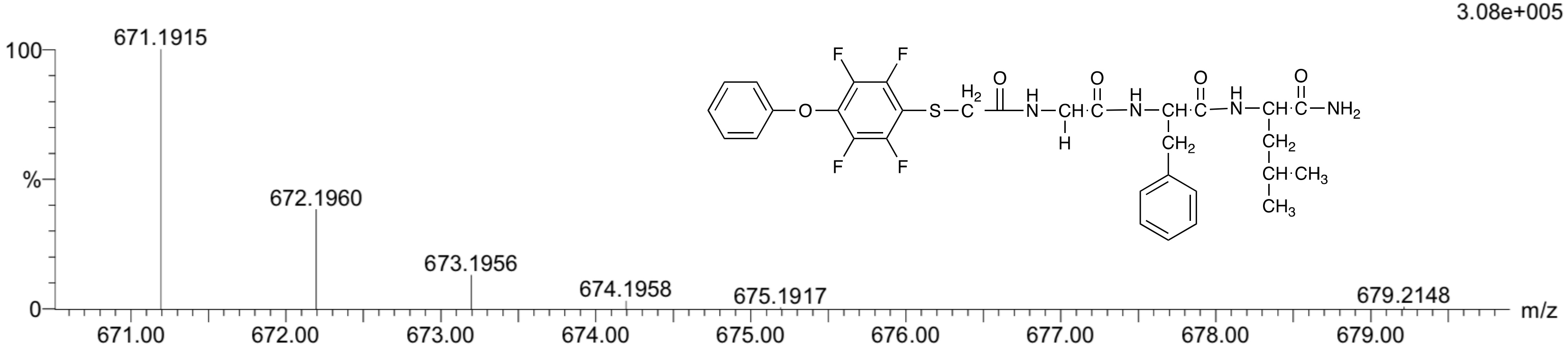

Minimum:				-1.5			
Maximum:		5.0	5.0	50.0			
Mass	Calc. Mass	mDa	PPM	DBE	i-FIT	i-FIT (Norm)	Formula
671.1915	671.1927	-1.2	-1.8	15.5	14.7	0.0	C31 H32 N4 O5 F4 Na S

LC of Entry **11,** Table **2**.

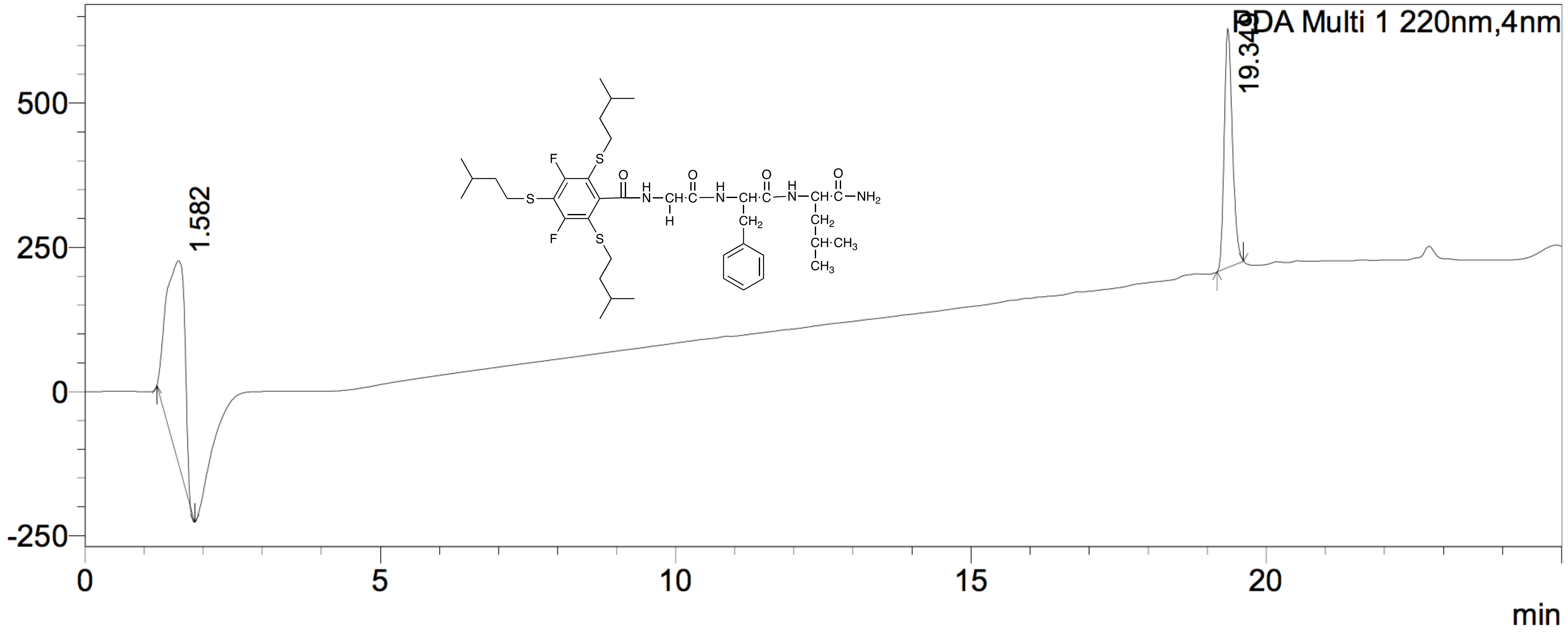

^{19}F NMR spectrum of Entry **11,** Table **2**.

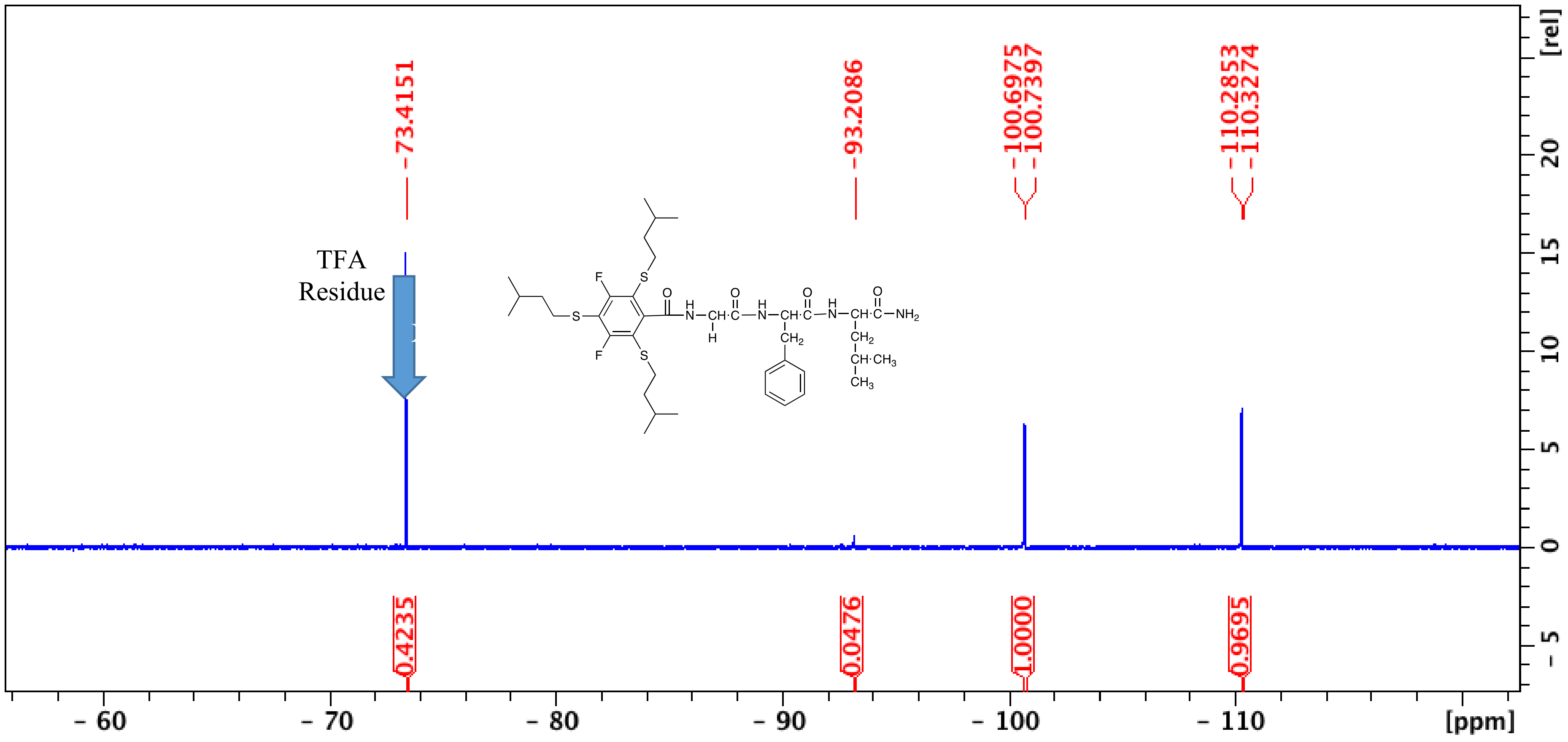

HRMS of Entry **11,** Table **2**.

Single Mass Analysis

Tolerance = 5.0 PPM / DBE: min = -1.5, max = 50.0
Element prediction: Off
Number of isotope peaks used for i-FIT = 2

Monoisotopic Mass, Even Electron Ions
32 formula(e) evaluated with 1 results within limits (up to 20 closest results for each mass)
Elements Used:
C: 35-40 H: 55-60 N: 0-5 O: 4-5 F: 0-2 Na: 1-1 S: 3-5
SF 2 40 (1.315) Cm (1:61)
TOF MS ES+

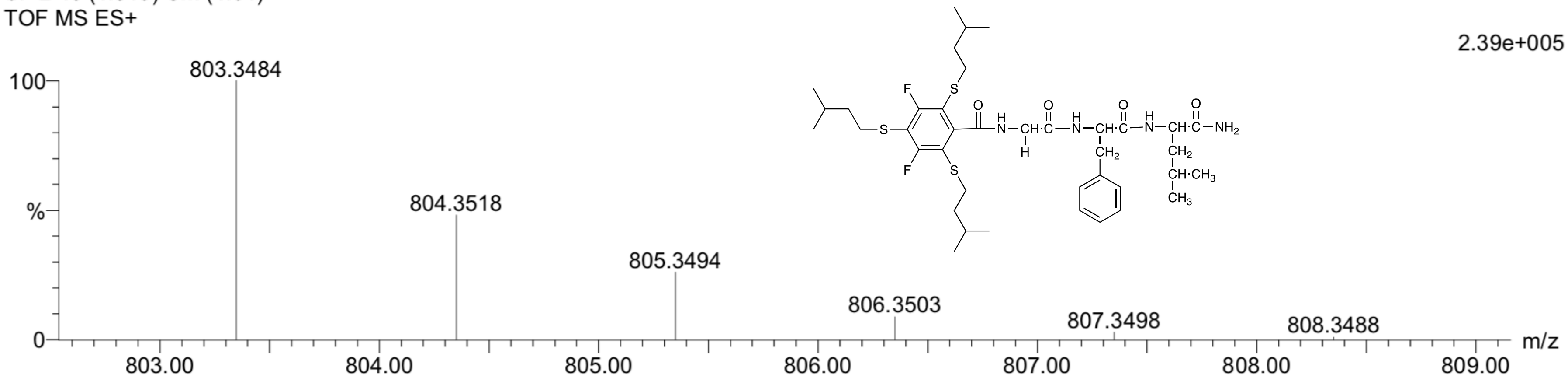

Minimum:				-1.5			
Maximum:		5.0	5.0	50.0			
Mass	Calc. Mass	mDa	PPM	DBE	i-FIT	i-FIT (Norm)	Formula
803.3484	803.3486	-0.2	-0.2	11.5	10.2	0.0	C39 H58 N4 O4 F2 Na S3

LC of Entry **12,** Table **2**.

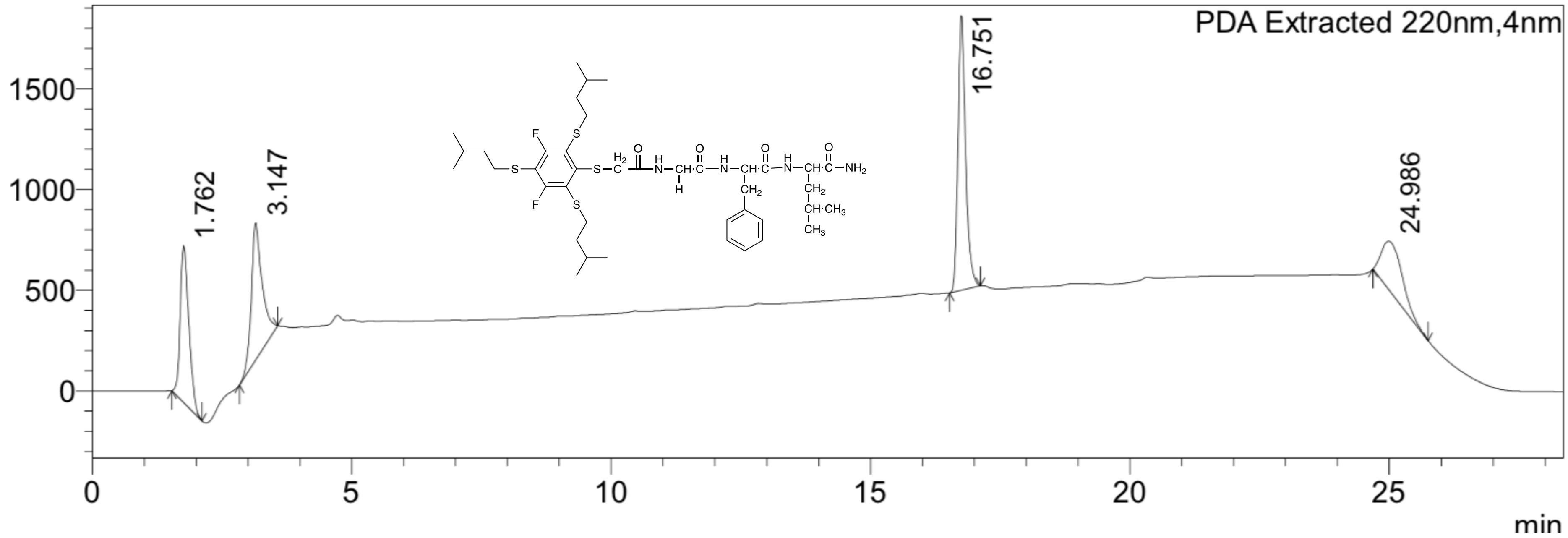

^{19}F NMR spectrum of Entry **12,** Table **2**.

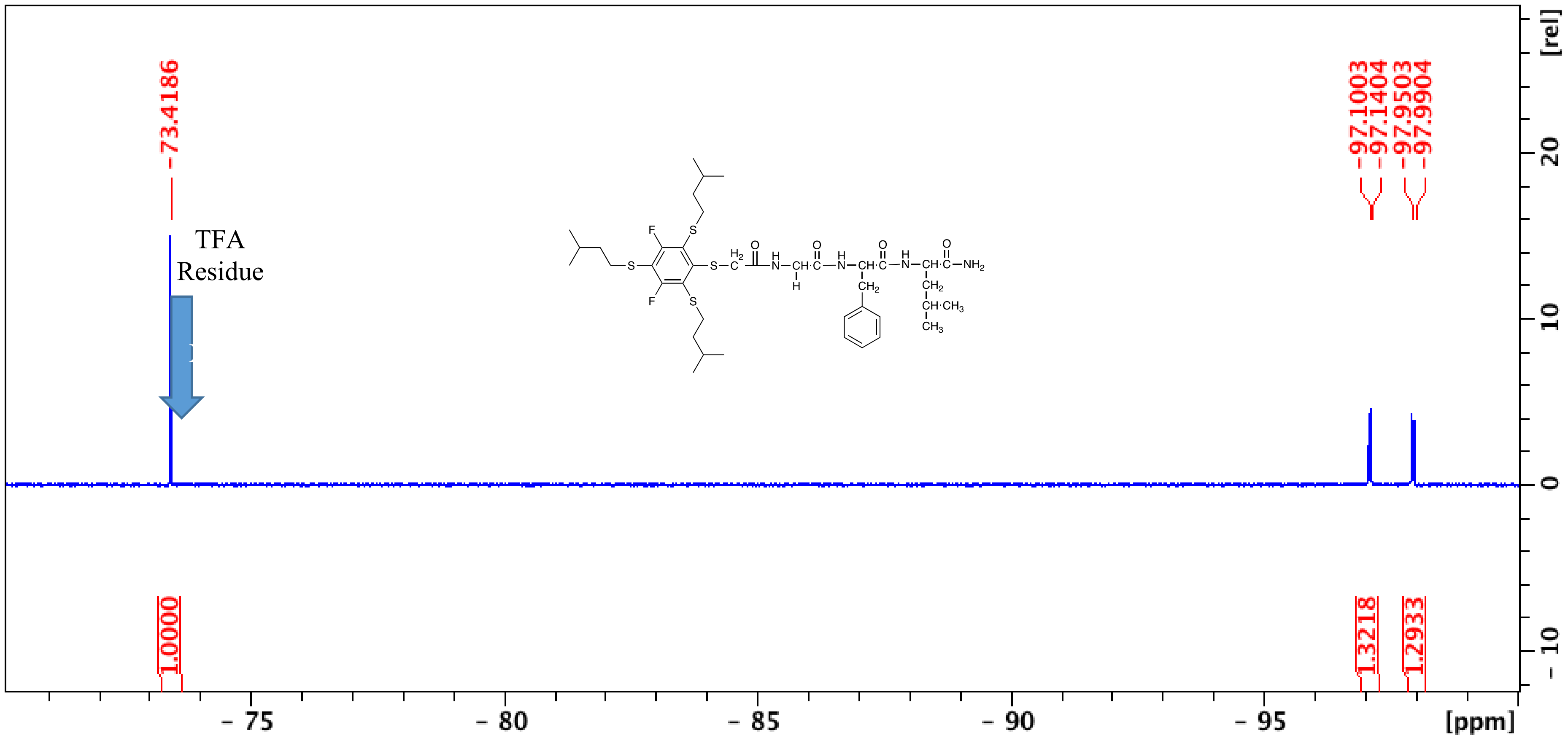

HRMS of Entry **12,** Table **2**.

Single Mass Analysis

Tolerance = 5.0 PPM / DBE: min = -1.5, max = 50.0
Element prediction: Off
Number of isotope peaks used for i-FIT = 2

Monoisotopic Mass, Even Electron Ions
54 formula(e) evaluated with 1 results within limits (up to 20 closest results for each mass)
Elements Used:
C: 40-45 H: 60-65 N: 0-5 O: 3-5 F: 1-2 Na: 1-1 S: 0-4
SS 2 10 (0.304)
TOF MS ES+

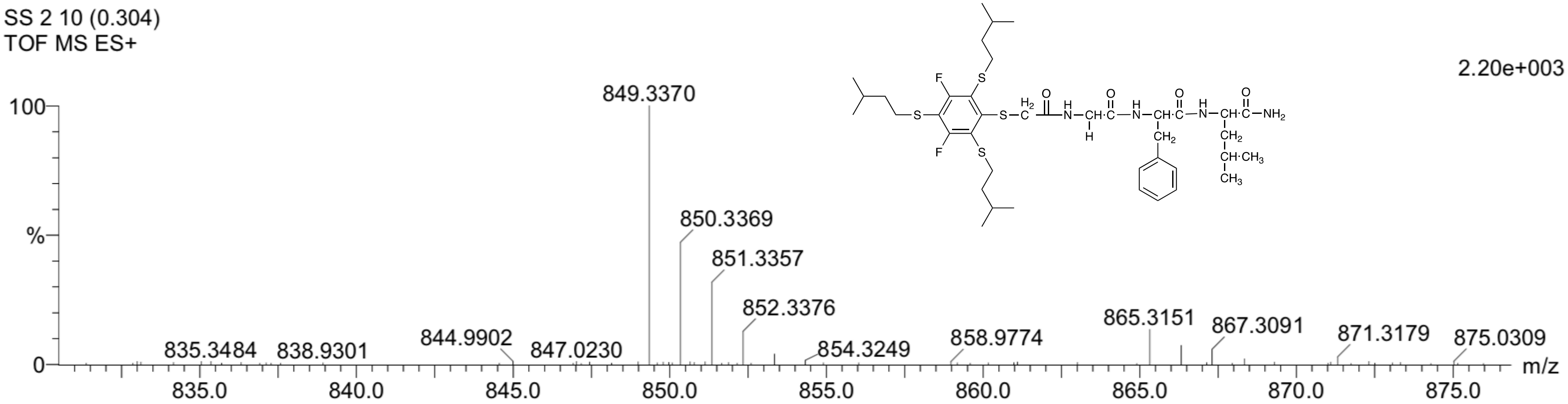

Minimum:				-1.5			
Maximum:		5.0	5.0	50.0			
Mass	Calc. Mass	mDa	PPM	DBE	i-FIT	i-FIT (Norm)	Formula
849.3370	849.3363	0.7	0.8	11.5	42.7	0.0	C40 H60 N4 O4 F2 Na S4

Entry **13,** Table **2**.

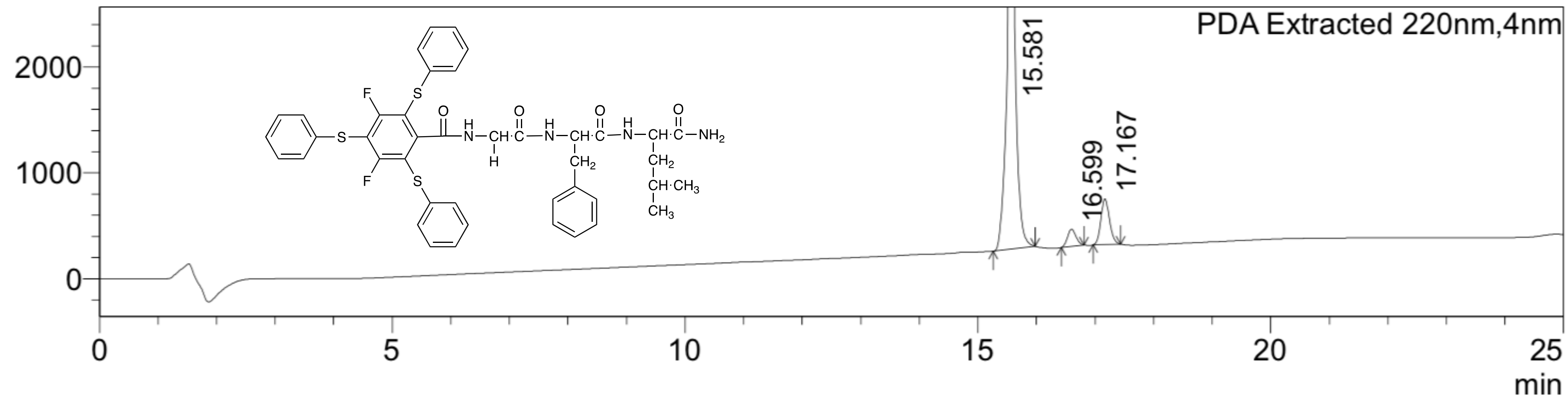

^{19}F NMR spectrum Entry **13,** Table **2**.

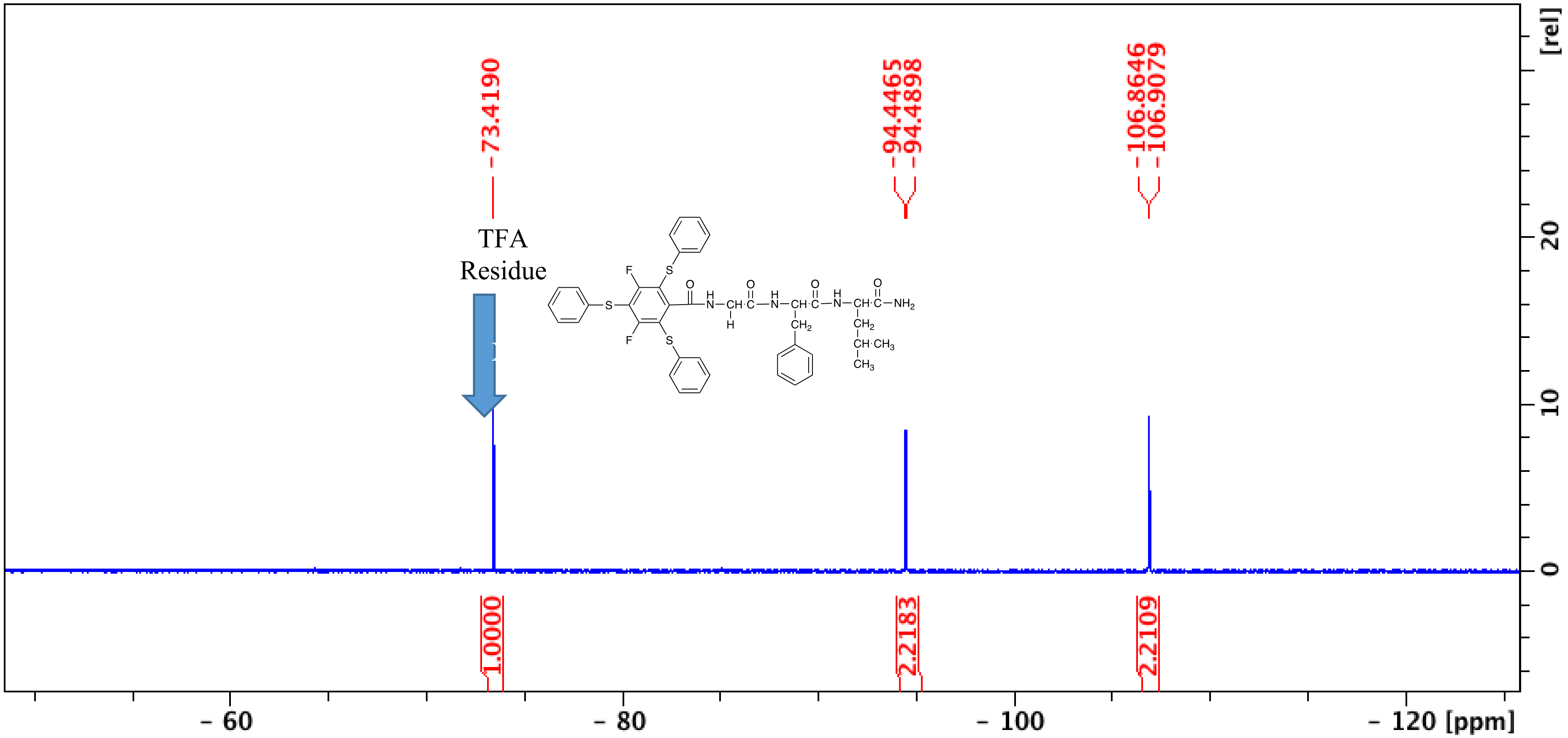

HRMS of Entry **13,** Table **2**.

Single Mass Analysis

Tolerance = 5.0 PPM / DBE: min = -1.5, max = 50.0
Element prediction: Off
Number of isotope peaks used for i-FIT = 2

Monoisotopic Mass, Even Electron Ions
172 formula(e) evaluated with 1 results within limits (up to 20 closest results for each mass)
Elements Used:
C: 40-45 H: 40-45 N: 0-5 O: 0-5 F: 0-2 Na: 1-1 S: 0-3
SF 7 60 (1.990) Cm (1:61)
TOF MS ES+

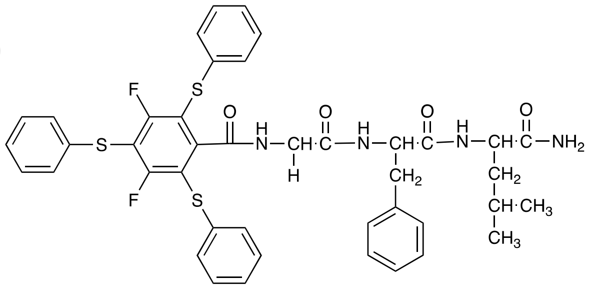

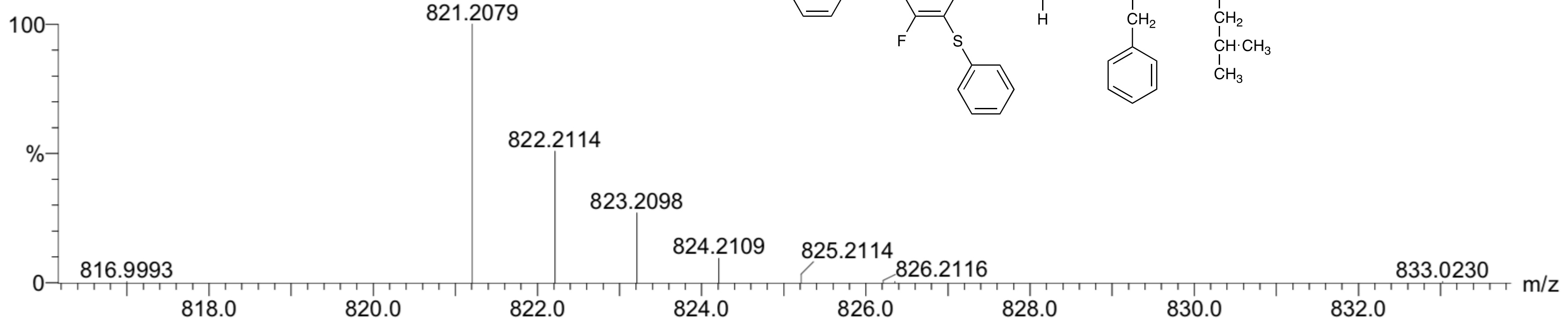

Minimum:				-1.5			
Maximum:		5.0	5.0	50.0			
Mass	Calc. Mass	mDa	PPM	DBE	i-FIT	i-FIT (Norm)	Formula
821.2079	821.2077	0.2	0.2	23.5	10.8	0.0	C42 H40 N4 O4 F2 Na S3

LC of Entry **14,** Table **2**.

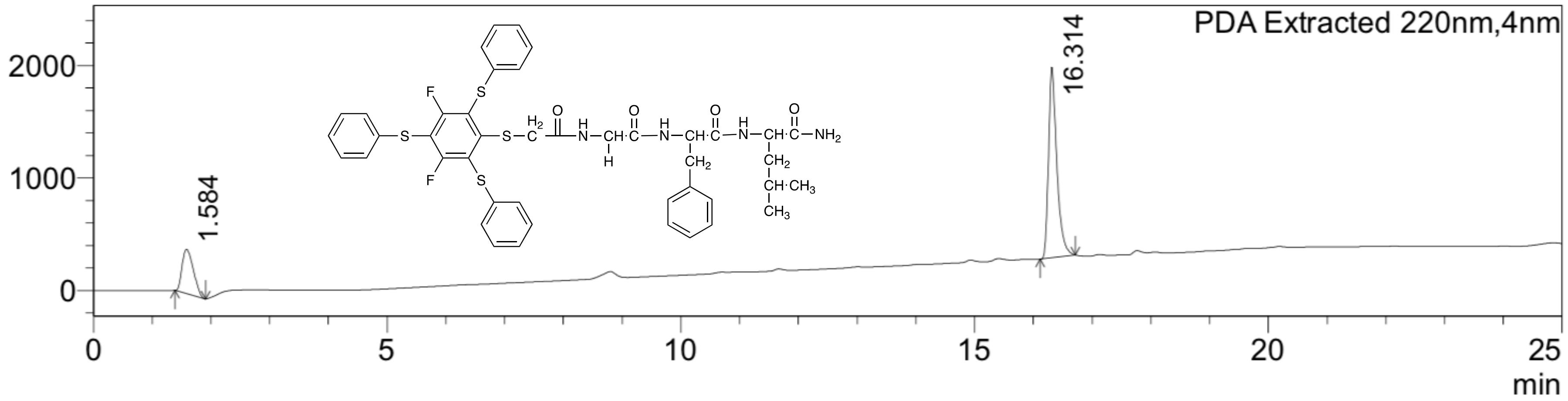

^{19}F NMR spectrum of Entry **14,** Table **2**.

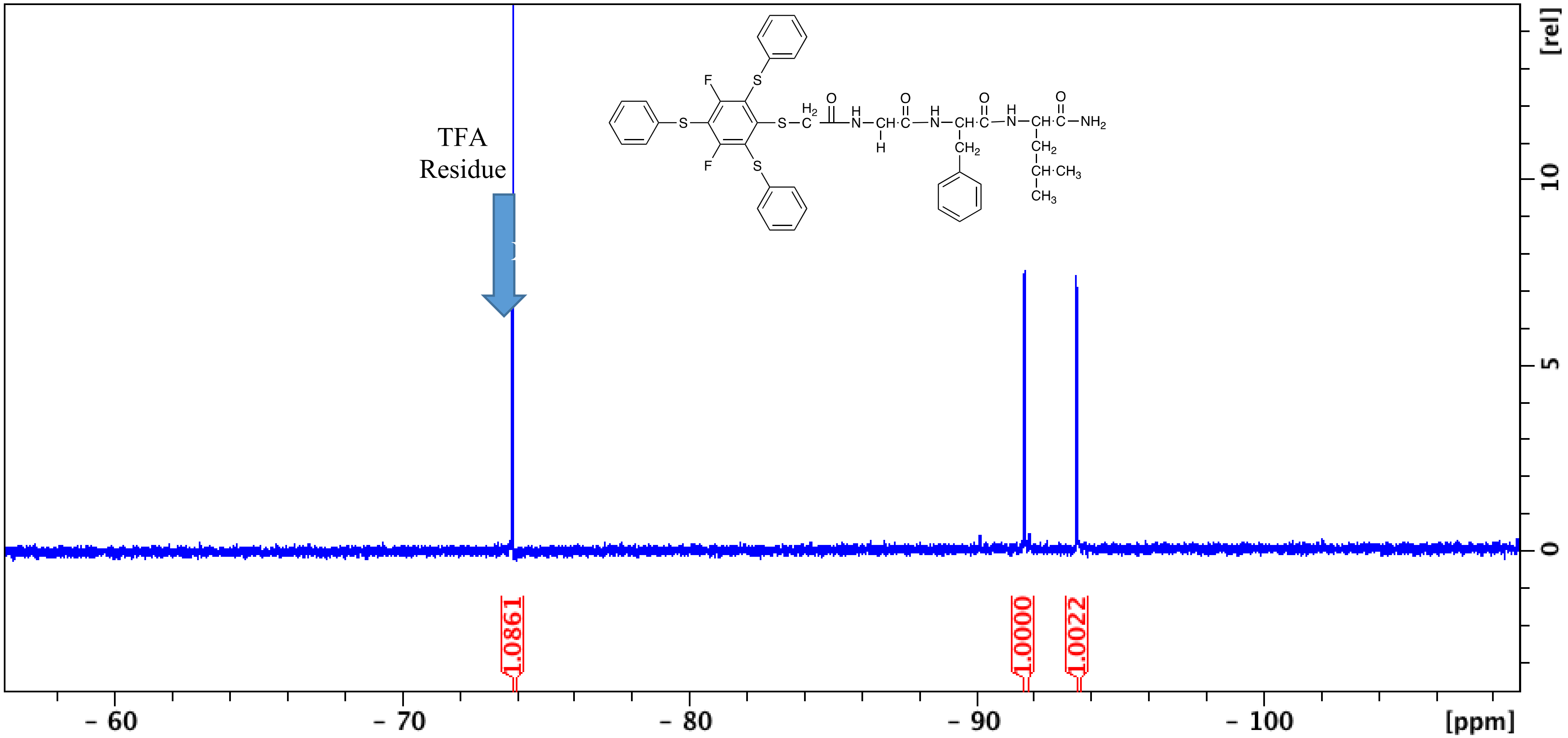

HRMS of Entry **14,** Table **2**.

Single Mass Analysis

Tolerance = 5.0 PPM / DBE: min = -1.5, max = 50.0
Element prediction: Off
Number of isotope peaks used for i-FIT = 2

Monoisotopic Mass, Even Electron Ions
311 formula(e) evaluated with 1 results within limits (up to 20 closest results for each mass)
Elements Used:
C: 40-45 H: 40-45 N: 0-5 O: 0-5 F: 0-2 Na: 1-1 S: 0-4
SS 6 2 (0.034) Cm (1:61)
TOF MS ES+

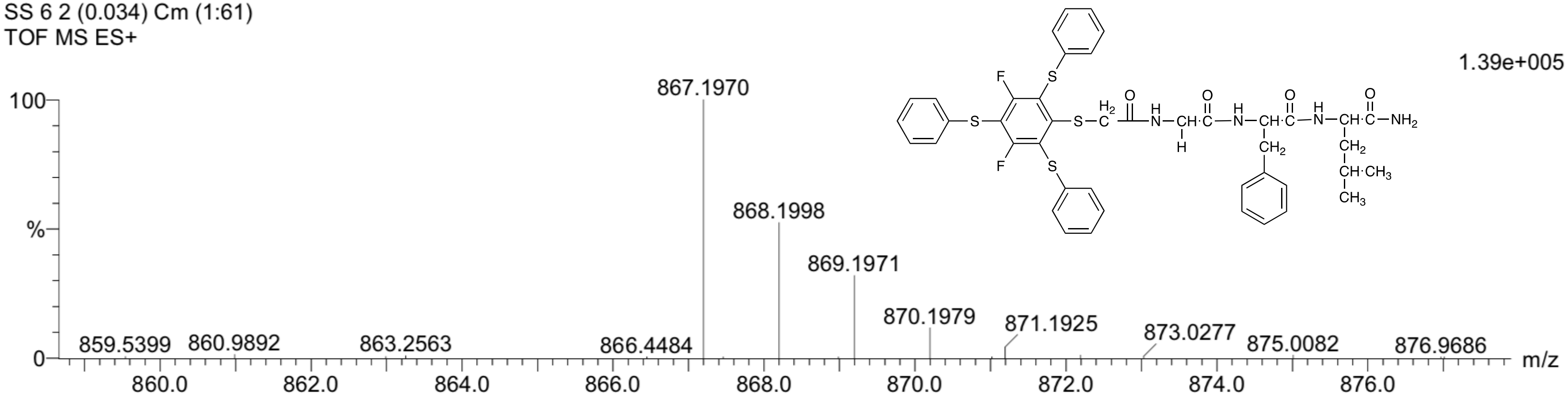

Minimum:				-1.5			
Maximum:		5.0	5.0	50.0			
Mass	Calc. Mass	mDa	PPM	DBE	i-FIT	i-FIT (Norm)	Formula
867.1970	867.1955	1.5	1.7	23.5	24.8	0.0	C43 H42 N4 O4 F2 Na S4

LCMS of Entry **11**, table **1** in the presence of DIEA as a base

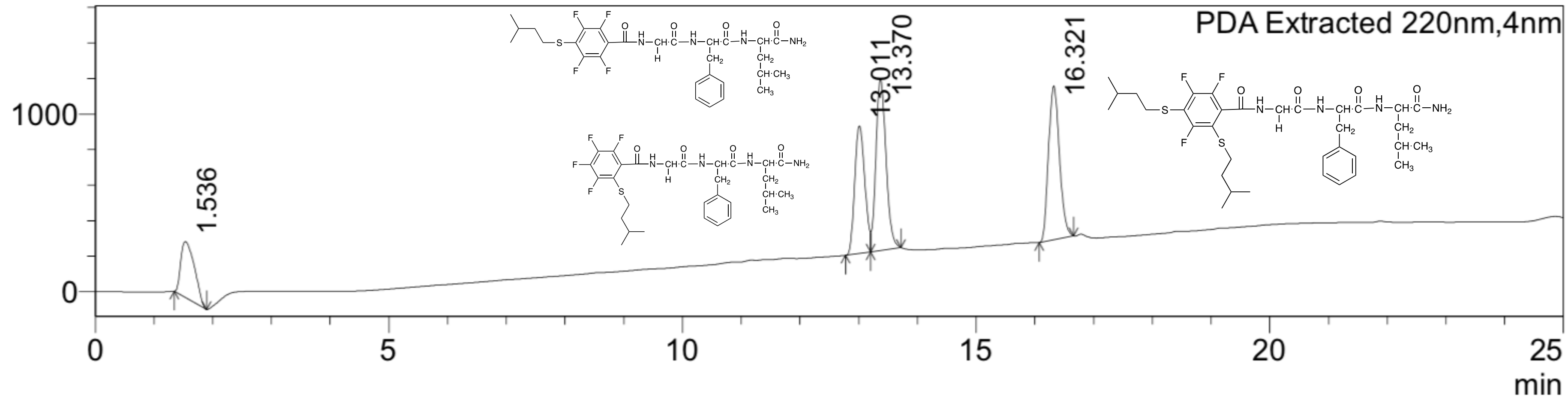

[19]NMR of Entry **11**, table **1** in the presence of DIEA as a base

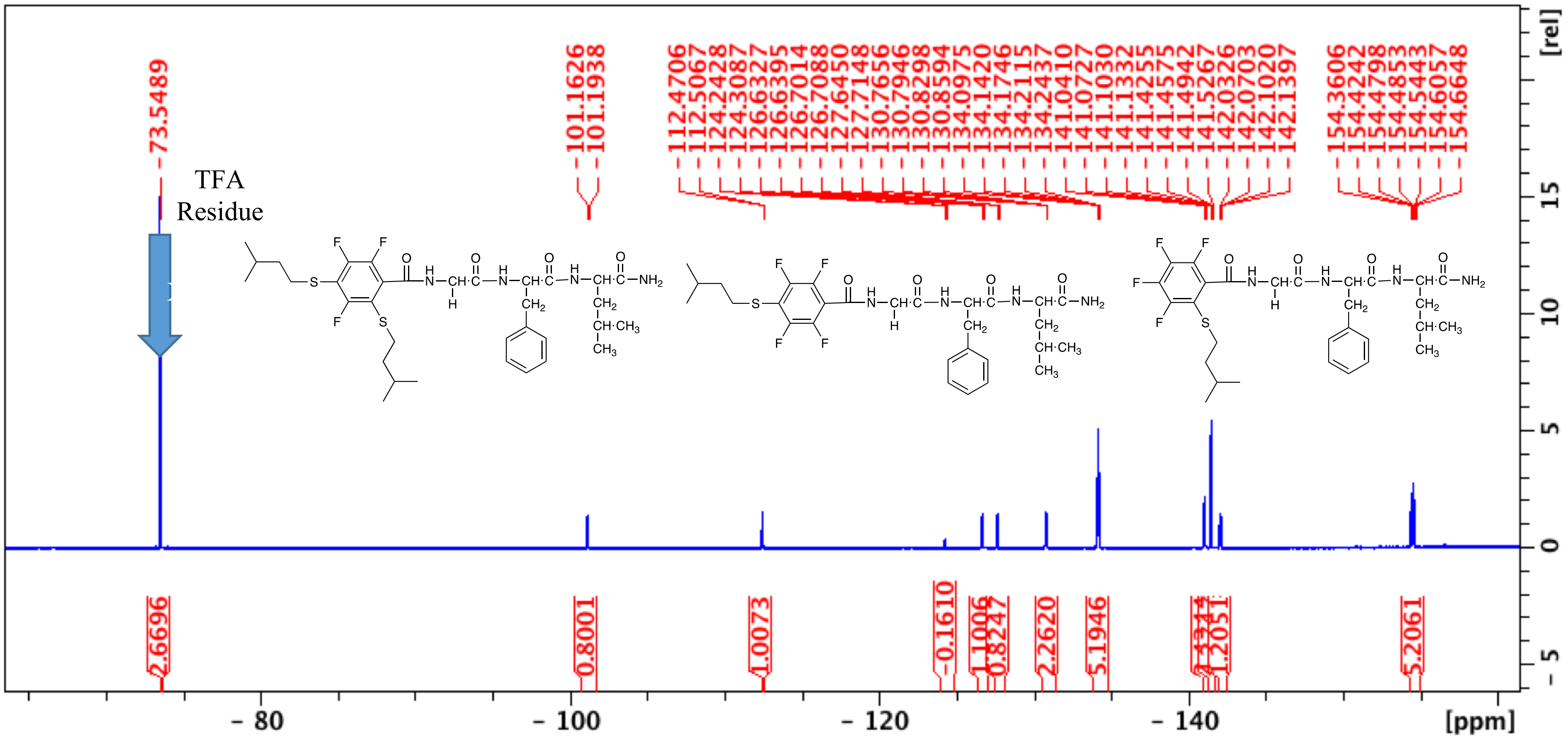
-73.5489
TFA
Residue
-101.1626
-101.1938
[rel]
[ppm]
- 80
- 100
- 120
- 140
2.6696
0.8001
1.0073
-0.1610
5.1946
2.2620
5.2061
